# Tax revenue lost due to illicit cigarettes in South Africa: 2002−2022

**DOI:** 10.1136/bmjopen-2023-077855

**Published:** 2024-03-14

**Authors:** Nicole Vellios, Corné van Walbeek

**Affiliations:** 1 Research Unit on the Economics of Excisable Products, School of Economics, University of Cape Town, Rondebosch, Western Cape, South Africa

**Keywords:** Health economics, Health policy, Behavior

## Abstract

**Abstract:**

**Objective:**

To estimate lost excise and value-added tax (VAT) revenue as a result of illicit cigarette trade from 2002 to 2022.

**Design:**

Using gap analysis, we estimated the number of illicit cigarettes by calculating the difference between the number of self-reported cigarettes (derived from nationally representative surveys) and the number of legal (tax-paid) cigarettes (derived from government sources) from 2002 to 2022. We then calculated the excise and VAT revenue that the government lost through illicit trade, taking into account that some people would have quit or reduced their consumption if cigarette prices had been higher (ie, tax paid).

**Setting:**

South Africa.

**Outcome measures:**

Illicit trade estimates and lost revenue estimates.

**Results:**

The illicit cigarette market comprised 5% of the market in 2009, peaked at 60% in 2021, and decreased to 58% in 2022. Accounting for the fact that some people would have reduced their consumption if cigarette prices had been higher (had the illicit marke not existed), the government lost R15 billion in excise revenue and R3 billion in VAT revenue in 2022. From 2002 to 2022, the government lost R119 billion (2022 prices) in excise and VAT revenue. The majority of the lost revenue occurred in the period 2010 to 2022, where R110 billion (2022 prices) in excise and VAT revenue was lost. A comprehensive sensitivity analysis indicates that the estimated lost revenue of R119 billion from 2002 to 2022 falls within the range of R65 billion to R130 billion (all 2022 prices).

**Conclusions:**

The South African government has been losing a significant amount of revenue by not receiving excise and VAT from all cigarettes consumed in South Africa. This trend is likely to continue if the government does not secure the supply chain from the point of production to the point of sale.

**STRENGTHS AND LIMITATIONS OF THIS STUDY**

- We provide estimates of illicit trade in South Africa covering more than two decades.
- We estimate lost excise and value-added tax revenue, taking into account that, if prices had been higher (ie, tax paid), some people would have quit smoking or would have reduced their consumption.
- The final computation of the size of the illicit market and the amount of lost tax revenue is an amalgam of many different estimates and assumptions, each of which is subject to a degree of uncertainty.

## Introduction

 Increasing excise taxes is the most effective tobacco control policy for reducing smoking prevalence.^1 2^ Reducing smoking prevalence decreases the substantial burden of disease caused by smoking, which was estimated to be around 25 700 premature deaths in 2016 among South Africans aged 35–74.^3^

British American Tobacco (BAT) and its predecessors (Rembrandt Tobacco Company) have dominated the South African tobacco landscape for many decades in the 20th century. In 2005, BAT still had a 91% market share.^4^ In subsequent years, other multinationals (mainly Philip Morris International and Japan Tobacco International) entered the market. From 2010, small manufacturers, based in South Africa and neighbouring countries, entered the market. They were presumably attracted by the very high profits made by the multinationals.^5^ Using the large excise tax increases as a pretext, the multinationals increased the retail price of cigarettes since the early 1990s, thus increasing their profit per cigarette.^6^ A large proportion of cigarette sales by the new entrants were sold at prices which did not even cover the excise tax (a practice that continues today). Instability at the South African Revenue Service (SARS), that started around 2014, resulted in a reduced capacity to collect excise taxes from cigarettes.

The illicit trade problem was further exacerbated by the 5 month ban on cigarette sales related to the COVID-19 pandemic in 2020.^7^ After the sales ban ended, a substantial proportion of smokers, who had previously smoked multinational brands, stayed with the non-multinational brands.^8^

National Treasury’s annual Budget Reviews provide an overview of the actual revenue (for previous financial years), predicted revenue (for the current financial year), and budgeted revenue (for the next financial year). National Treasury’s predictions of excise revenue from cigarettes and cigarette tobacco have become less accurate over time (online supplemental figure S1). From 2003/2004 to 2009/2010, actual revenue was close to budgeted revenue, but subsequently was often well below budgeted revenue.^9^ The decrease in excise revenue, if unaccompanied by a decrease in smoking prevalence, indicates a growing illicit market.

Existing evidence shows that illicit local manufacturing is the major source of the gap between self-reported consumption and registered tax-paid sales in South Africa. This evidence includes a 2019 book by an ex-SARS official,^10^ a thorough qualitative survey of the illicit market in South Africa and Zimbabwe,^11^ investigative journalism,^12^ and localised surveys of townships.^13^

In this paper, we provide estimates of illicit trade from 2002 to 2022, updating a 2019 paper that estimated the size of the illicit market from 2002 to 2017.^14^ An update is necessary because the illicit market has grown substantially since 2017, largely owing to the 5 month COVID-19 cigarette sales ban in 2020.^7^ In addition to the updated estimates of the size of the illicit market, our main contribution is to provide estimates of lost tax revenue. There are no estimates of lost tax revenue in the peer-reviewed literature. To the best of our knowledge, this is the first paper that acknowledges that there would be a reduction in aggregate cigarette consumption if illicit trade had not existed. We take into account the possibility that, if prices had been higher (ie, tax paid), some people would have quit smoking or would have reduced their consumption, because cigarettes would have been unaffordable. This is a substantial improvement to estimates of lost tax revenue calculated by the tobacco industry, who simply multiply the estimated number of illicit cigarettes by the tax per cigarette. For example, in a 2023 article^15^ BAT’s estimate of lost excise revenue for 2022 was quoted as R24 billion, which was calculated as their estimate of the illicit market (1.22 billion packs of 20 cigarettes) multiplied by the excise tax (R19.82 per pack). The practice of accounting for a decrease in consumption, had illicit trade not existed, should be applied to other countries where illicit cigarettes are sold at prices well below the prices of legal cigarettes. This is a novel contribution to the illicit trade literature.

## Methods

### Data

Smoking behaviour data were drawn from nationally representative data sets. Smoking prevalence data from 2002 to 2015 for those aged 15+ were drawn from the All Media and Products Survey (AMPS).^16^ Smoking prevalence for 2021 was drawn from the 2021 Global Adult Tobacco Survey (GATS),^17^ which provides the most recent nationally representative data for smoking prevalence. Although the granular data have not yet been released, a factsheet indicates *current* cigarette smoking prevalence (which includes manufactured cigarettes, hand-rolled cigarettes, and kreteks) of 23.9% among those aged 15+.^17^ The lead researcher on GATS 2021 data collection indicated that current manufactured cigarette smoking prevalence was 23.4%. As part of a sensitivity analysis, we also used a *daily* manufactured cigarette prevalence of 20% in 2021. Smoking intensity data from 2002 to 2011 were drawn from AMPS. Smoking intensity estimates for 2020 were drawn from the National Income Dynamics Study (NIDS): Coronavirus Rapid Mobile Survey (2020).^8^

Annual population data (age 15+) for the period 2002 to 2021 were obtained from the United Nations (UN) population database.^18^ Anomalies in the data from 2014 to 2019 required us to smooth the data between those years, where we assumed a constant population growth rate. At the time the analysis was done, the UN had not yet released the 2022 population figures, which we estimated by applying the average population growth rate of the past 10 years, namely 1.23%, to the 2021 population figure. The amount of excise revenue received from locally produced cigarettes was obtained from annual Budget Reviews published by the National Treasury.^9^ Excise tax rates, which apply to locally made and imported cigarettes, were also drawn from the annual Budget Reviews. The number of imported cigarettes was obtained from the Department of Trade, Industry and Competition (DTIC).^19^ The imported cigarette data for 2002 to 2015 were smoothed using techniques described elsewhere.^14^ To adjust for inflation, all data are presented in constant 2022 prices, using Consumer Price Index (CPI) data from Statistics South Africa to inflate the nominal prices of previous years.^20^

To estimate the decrease in consumption if the illicit market had not existed, we conducted an analysis using cigarette price data from NIDS wave 5 conducted in 2017.^21^

### Patient and public involvement

It was not appropriate or possible to involve patients or the public in the design, or conduct, or reporting, or dissemination plans of our research.

### Estimating the number of illicit cigarettes in the market

We used a gap analysis approach to estimate the number of illicit cigarettes in the market. This approach has been described in detail elsewhere,^14 22^ and is only briefly explained here. Illicit trade is measured as the gap between self-reported cigarette consumption and tax-paid cigarette sales.

Self-reported annual consumption is the product of four components: (1) the size of the adult population (aged 15+), (2) smoking prevalence, (3) average number of cigarettes smoked per day, and (4) 365 days per year. Because there is no more up-to-date prevalence statistic than the 2021 GATS estimate, we used a smoking prevalence of 23.4% for 2022. Smoking prevalence data for 2016–2020 were interpolated. For 2020, we reduced the smoking prevalence by 4%. This figure is derived from the fact that the 20 week sales ban (40% of the year) temporarily reduced the number of smokers by approximately 10% (40% x 10%=4%).^23^

The average number of cigarettes smoked per day was obtained from AMPS for the period 2002 to 2011, and from NIDS-CRAM for 2020. Estimates of smoking intensity for 2012 to 2019 were interpolated. The number of cigarettes smoked per day was estimated at 9.1 in 2011 and 8.8 in 2020. For 2021 and 2022, we kept the number at 8.8 cigarettes per day. We applied a 3 year-centred moving average to smooth self-reported consumption estimates for the years 2003 through 2021. For the first and last years in the series, the moving average is based on only 2 years (2002 and 2003 for 2002, and 2021 and 2022 for 2022).

To account for the under-reporting of cigarette consumption, which is common in surveys, we uplifted the reported numbers by 5% for two reasons: (1) it ensures that the volume of illicit trade would not be less than zero in any year since negative illicit trade is nonsensical, and (2) we anchored the 5% to obtain illicit trade estimates conversant with the existing body of literature on illicit trade estimates in South Africa.^5 13 24 25^ The calculation for 5% under-reporting is x/0.95, where x is self-reported consumption. In 2022, accounting for an under-reporting of 5%, the number of self-reported cigarettes was 33.7 billion sticks.

To create confidence intervals around annual self-reported cigarette consumption, Stata V.16.0 was used to bootstrap the point estimate of the product of individual smoking status (which is used to determine smoking prevalence in the population) and smoking intensity. Bootstrapping obtains a valid SE by computing the estimate from different random samples drawn from the original data.^26^ This method has been used before by Paraje.^27^

Smoking status is a discrete variable (1: smoker, 0: non-smoker), while cigarette consumption is a continuous variable. Respondents who reported that they smoked, but did not report the number of cigarettes they smoked per day, were assigned the mean value of smoking intensity in that year. Where we did not have smoking intensity data (AMPS 2012−2015), we only bootstrapped the number of smokers, assuming cigarette smoking intensity estimates as presented in table 1.

**Table 1:** Background data to estimate the number of illicit cigarettes in the market (95% CIs in brackets)

Year	Total adult population (million)	Smoking prevalence(%)	Number of smokers (million)	Average daily smoking intensity (sticks)	Self-reported consumption (billion sticks)	Self-reported consumption, 3-year moving average, accounting for 5% under-reporting (billion sticks)	Annual tax-paid consumption, local and imported (billion sticks)	Illicit trade as a % of total market
	(1)	(2)	(3)	(4)	(5)	(6)	(7)	(8)
2002	31.8	25.2(24.7 to 25.6)	8.0(7.8 to 8.2)	9.1(8.9 to 9.2)	26.5(25.7 to 27.3)	27.6(26.7 to 28.4)	26.1	5.2(2.2 to 8.1)
2003	32.6	23.7(23.2 to 24.3)	7.7(7.6 to 7.9)	9.2(9.0 to 9.3)	25.9(25.0 to 26.7)	27.6(26.8 to 28.5)	26.0	5.9(2.8 to 8.7)
2004	33.3	24.5(23.9 to 25.0)	8.1(8.0 to 8.3)	8.9(8.7 to 9.1)	26.4(25.6 to 27.3)	27.7(26.8 to 28.6)	25.6	7.3(4.2 to 10.2)
2005	34.0	24.3(23.7 to 24.8)	8.2(8.1 to 8.4)	8.8(8.7 to 9.0)	26.6(25.7 to 27.5)	28.1(27.1 to 29.0)	25.7	8.5(5.3 to 11.5)
2006	34.6	24.6(24.0 to 25.1)	8.5(8.3 to 8.7)	8.7(8.6 to 8.9)	27.1(26.1 to 28.0)	28.7(27.7 to 29.7)	26.1	9.0(5.7 to 12.0)
2007	35.2	25.2(24.7 to 25.8)	8.9(8.7 to 9.1)	8.7(8.5 to 8.9)	28.2(27.2 to 29.2)	28.7(27.6 to 29.8)	26.7	7.2(3.6 to 10.6)
2008	35.7	23.5(22.9 to 24.1)	8.4(8.2 to 8.6)	8.7(8.5 to 8.8)	26.5(25.4 to 27.7)	28.7(27.6 to 29.9)	27.1	5.5(1.7 to 9.1)
2009	36.3	23.3(22.8 to 23.8)	8.5(8.3 to 8.7)	8.8(8.6 to 8.9)	27.1(26.0 to 28.2)	27.4(26.3 to 28.5)	26.1	4.8(0.8 to 8.4)
2010	36.9	19.8(19.3 to 20.3)	7.3(7.1 to 7.5)	9.2(9.0 to 9.4)	24.5(23.6 to 25.5)	26.9(25.9 to 28.0)	23.6	12.5(8.9 to 15.8)
2011	37.5	20.2(19.7 to 20.7)	7.6(7.4 to 7.7)	9.1(8.9 to 9.3)	25.1(24.1 to 26.1)	26.7(25.7 to 27.7)	22.8	14.7(11.4 to 17.9)
2012	38.0	21.1(20.6 to 21.6)	8.0(7.8 to 8.2)	*9.1*	26.5(25.6 to 27.5)	27.5(26.5 to 28.5)	23.1	16.1(12.9 to 19.0)
2013	38.6	21.0(20.5 to 21.5)	8.1(7.9 to 8.3)	*9.0*	26.7(25.7 to 27.6)	28.0(27.0 to 29.0)	22.2	20.8(17.9 to 23.5)
2014	39.0	20.8(20.3 to 21.3)	8.1(7.9 to 8.3)	*9.0*	26.6(25.7 to 27.6)	28.6(27.6 to 29.5)	23.2	18.9(16.0 to 21.6)
2015	39.5	21.7(21.2 to 22.2)	8.6(8.4 to 8.8)	*9.0*	28.1(27.1 to 29.0)	29.3(27.8 to 29.8)	23.0	21.3(17.9 to 23.4)
2016	40.0	*22.0*	8.8	*9.0*	28.7	30.2	22.0	27.1
2017	40.5	*22.3*	9.0	*8.9*	29.3	30.8	19.6	36.4
2018	40.9	*22.5*	9.2	*8.9*	29.9	31.5	20.7	34.1
2019	41.4	*22.8*	9.5	*8.9*	30.5	31.7	19.0	39.9
2020	41.9	*22.2*	9.3	8.8(7.7 to 9.9)	29.9	32.4	13.7	57.7
2021	42.4	23.4(20.8 to 26.2)	9.9(8.8 to 11.1)	*8.8*	31.8	33.0	13.3	59.7
2022	42.9	*23.4*	10.0	*8.8*	32.2	33.7	14.3	57.6

Smoking prevalence and smoking intensity numbers in italics are interpolated of extrapolated. The relatively wider CIs for 2020 and 2021 is due to smaller samples. The sample sizes of successful interviews for the AMPS data ranges from a minimum of 20 377 (in 2008) to a maximum of 29 458 (in 2002), while the sample sizes of successful interviews is 6130 for NIDS-CRAM 2020 and 6311 for GATS 2021.

Since the bootstrap command does not allow weights to be accounted for using the svy command, we multiplied smoking*intensity by the weight variable before bootstrapping. All missing values were dropped before running the bootstrap command (failing to do so resulted in error messages). We specified 1000 repetitions in the bootstrap command.

Once the point estimate was obtained, it was multiplied by 365, and by the total number of respondents in each respective survey. For example, in AMPS 2002, annual self-reported consumption was 26.5 billion cigarettes (2466 cigarettes per day (bootstrap point estimate) * 365 (days per year) * 29 458 (number of respondents)). To obtain the 95% CIs for annual self-reporting cigarette consumption, the same formula was applied, using the lower and upper bound estimates.

Tax-paid sales volumes for locally produced cigarettes were estimated by dividing excise tax revenue by the excise tax per pack. For example, in the 2022/2023 financial year, the government indicated that it expected to collect R10.92 billion in excise taxes from locally produced cigarettes and cigarette tobacco. R10.92 billion is the ‘revised estimate’ as presented in the 2023 budget; ‘actual collection’ for 2022/2023 will only be presented in the 2024 budget. The excise tax for a pack of 20 cigarettes was R19.82. Around 551 million locally produced packs were sold on the legal market (R10.92 billion/R19.82) in 2022/2023. Annualising this number to the calendar year 2022 (taking 3/4 of the volume in the financial year 2022/2023 and 1/4 of the volume in the previous financial year) gives 532 million packs or 10.64 billion sticks. Adding to this, the 3.64 billion imported cigarettes^19^ results in a total legal (ie, tax-paid) cigarette market of 14.28 billion sticks in 2022.

The number of illicit sticks in 2022 is estimated at 19.4 billion sticks (ie, 33.7 billion self-reported cigarettes less 14.3 billion tax-paid cigarettes).

### Estimating lost excise and VAT revenue, accounting for the law of demand

The amount of lost excise tax revenue is not simply the number of illicit cigarettes multiplied by the excise tax. If the illicit market had not existed, fewer cigarettes would have been consumed, because some people would have been unable to afford tax-paid cigarettes. The quantity by which cigarette consumption is expected to have decreased depends primarily on the price elasticity of demand, which in turn is derived from the law of demand. We use the phrase ‘accounting for the law of demand’ to describe this effect.

In order to quantity this effect, we used data from NIDS wave 5 of 2017.^21^ In wave 5, as in the other NIDS waves, respondents were asked whether they smoked cigarettes or not, and, if they did, their daily consumption of cigarettes. In wave 5, respondents were asked about their most recent purchase of cigarettes (eg, the number of cigarettes purchased, the type of packaging, and the amount paid). From this data, we calculated the price paid per cigarette pack.

Of the 25 075 adults surveyed in NIDS wave 5, 4224 (19.3% based on weighted data) indicated that they smoked cigarettes. Of these, 3509 (83%) respondents provided enough information for us to derive the price paid per cigarette. In cases where the derived prices were nonsensical, we followed the data-cleaning conventions described in Appendix 1 of Van der Zee et al.^5^

To separate legal from illegal cigarettes, we estimated a price threshold of R23.00 in 2017, calculated as follows: R14.04 (annualised excise tax) + R2.50 (manufacturing cost) + R2.33 (wholesale and retail margins and distribution) + R1.31 (manufacturer profit) + R2.82 (14% VAT on the sum of the different components).

Annual excise taxes were obtained from National Treasury’s Budget Reviews.^9^ The manufacturing cost of R2.50 is based on personal communication between the second author and a representative of a multinational tobacco company on 20 November 2018. Wholesale and retail margins and distribution are set at 14% of the sum of excise tax plus manufacturing cost. This number was confirmed in a discussion between the second author and an industry representative, and can be calculated from the numbers given in a media article that extensively quotes the tobacco industry.^15^ This wholesale and retail mark-up percentage is applied to all years. The minimum profit margin of R1.31 in 2017 corresponds closely to the real value of the profit margin quoted by the industry.^15^ Any sale at a retail price of less than R23.00 indicates that taxes were not paid.

The common price points in the NIDS 2017 data set are R10, R20, R30, and R40 per pack. Online supplemental figure S2 illustrates the distribution of reported cigarette prices for a pack of 20 cigarettes in 2017 (nominal prices).

For each weighted observation where the reported price is less than the threshold retail price of R23.00 per pack, we calculated the expected decrease in consumption, should the price be increased to that level. This decrease depends on the price elasticity of demand. We used a price elasticity of −0.6, as this is frequently found in developing countries,^28^ including South Africa.^29 30^ As a sensitivity analysis, we also use price elasticities of −0.4 and −0.8. Since the prices of some illicit cigarettes were extremely low in 2017 (R10 per pack), an increase to R23.00 would have been substantial. In cases like this, the arc formula for the price elasticity of demand should be used to estimate the impact of the price change on consumption.^31^ It is specified as:


(1) $$\varepsilon_{p}=\left(\frac{Q_{2}-Q_{1}}{P_{2}-P_{1}}\right)*\left(\frac{P_{1}+P_{2}}{Q_{1}+Q_{2}}\right)$$

After the price change, the new quantity (*Q*_2_) is calculated as:


(2) $$Q_{2}=Q_{1}\left[1+\varepsilon_{p}\left(\frac{P_{2}-P_{1}}{P_{1}+P_{2}}\right)\right]/\left[1-\varepsilon_{p}\left(\frac{P_{2}-P_{1}}{P_{1}+P_{2}}\right)\right]$$

The quantity of cigarettes that are ‘lost’ (ie, not bought) because of the price increase is the difference between *Q*_1_ and *Q*_2_. This difference is expressed as a proportion of *Q*_1_.

For each year from 2002 to 2022, we applied the proportion estimated for 2017, because we only had granular price data for 2017. GATS 2021 is the only other data set that asked about prices at the individual level, but the raw data are not publicly accessible (as at January 2024).

To estimate lost excise revenue, we multiplied the annualised excise tax per pack by the number of illicit cigarettes, accounting for the law of demand. To estimate the lost VAT revenue, we first estimated the total VAT per pack, and then multiplied this by the number of illicit cigarettes, also accounting for the law of demand. VAT of 14% was applied for the years 2002 to 2016, 14.75% in 2017 (14% up to 31 March, 15% thereafter), and 15% from 2018 to 2022. The base value for the VAT in each year is the sum of the manufacturing cost, manufacturer’s profit, excise tax, and the wholesale and retail margins. Manufacturers’ costs and profit were adjusted by the CPI, such that the real value remained constant over time. Wholesale and retail margins and distribution were set at 14% of the sum of the excise tax and manufacturers’ costs.^15^

Results were computed using Stata V.16 and Microsoft Excel.

## Results

### Estimating the number of illicit cigarettes in the market

Annual self-reported consumption, the product of total adult population, smoking prevalence, daily smoking intensity and 365 days, was 26.5 billion sticks in 2002, increasing to 32.2 billion sticks in 2022 (table 1 column 5). To smooth the data, a 3 year-moving average was applied to column 5, and the data were also upscaled by 5% to account for under-reporting in survey data. This results in a total market of 27.6 billion sticks in 2002 and 33.7 billion sticks in 2022 (column 6).

**Figure 1:**
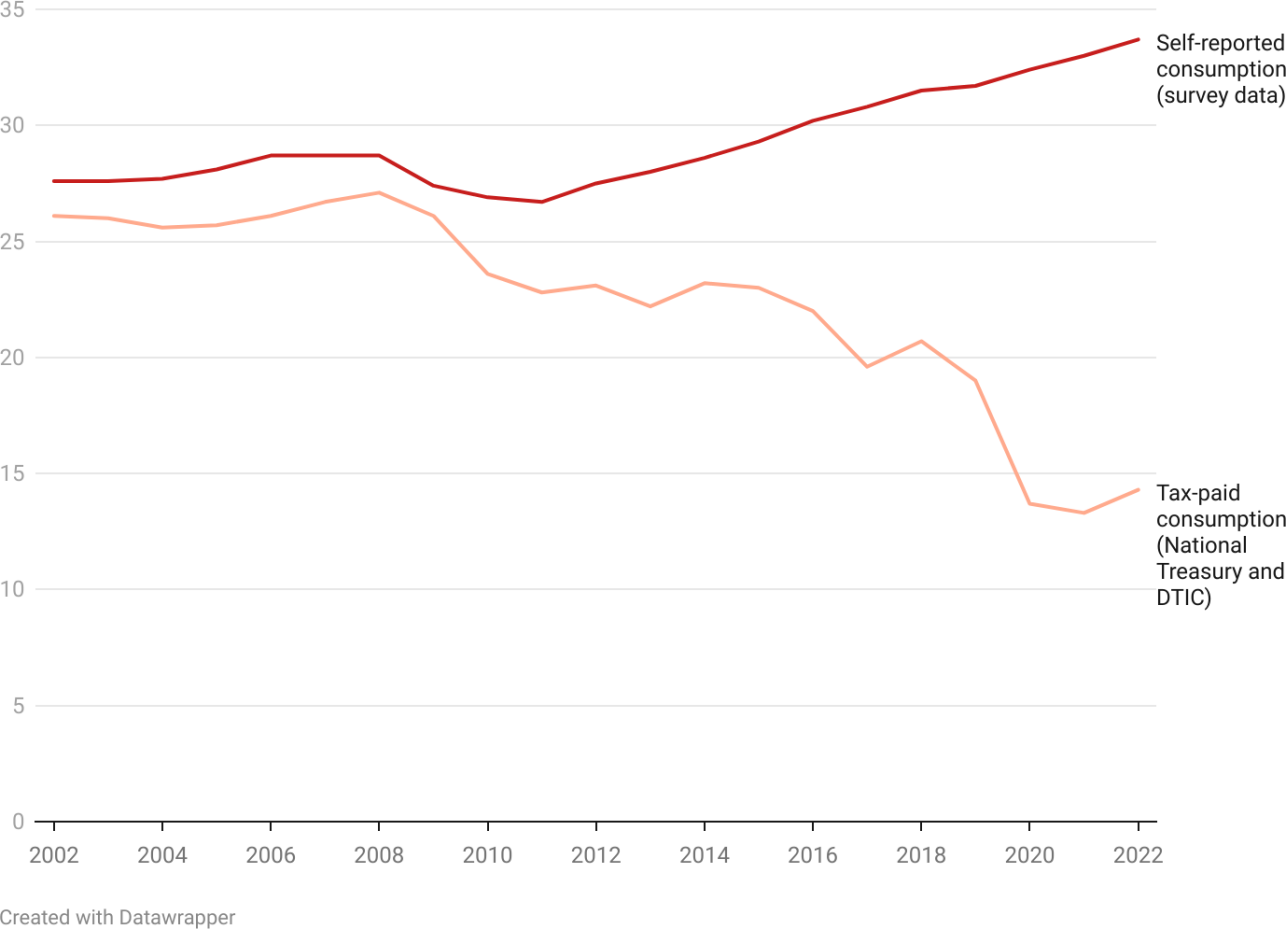
The widening gap between self-reported consumption and tax-paid consumption (billions of cigarette sticks).

**Figure 2:**
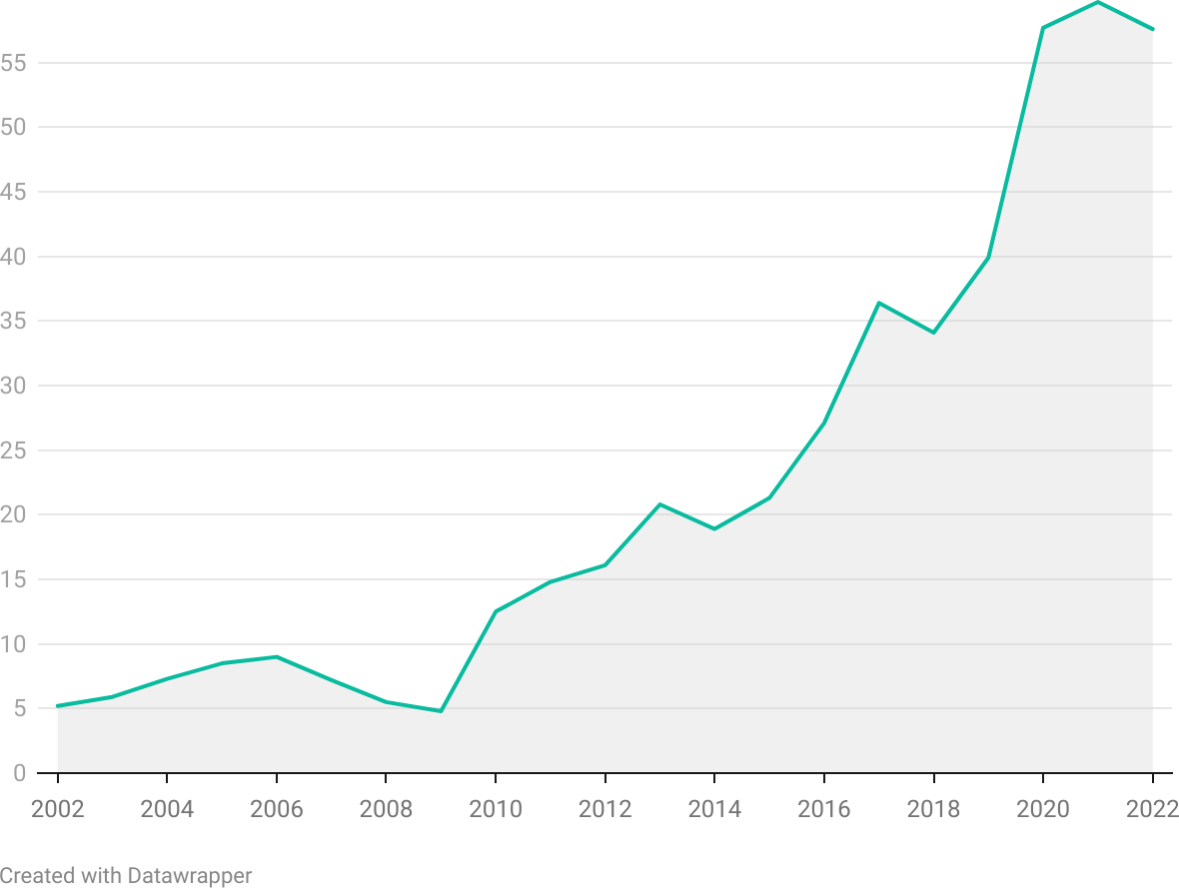
Percentage of the illicit trade in the total market.

The number of illicit sticks, defined as the gap between self-reported consumption (column 6) and registered sales (column 7), was less than 5 billion from 2002 to 2012, after which it increased rapidly, reaching 19.7 billion sticks in 2021. See figure 1 for a graphical depiction of the the widening gap. The illicit market percentage (column 8) is volume of illicit cigarettes, expressed as a percentage of self-reported consumption (column 6). Illicit trade is estimated to have been around 4.8% in 2009, increasing to 59.7% in 2021, and decreasing to 57.6% in 2022 (figure 2).

### Estimating lost revenue, accounting for the law of demand

In 2017, 32.8% of cigarettes (9.7 billion cigarettes) in the NIDS data set were sold at a retail price of R23 or less per pack. To illustrate the principle of the impact of the hypothesised price increase, consider the 2.6 billion cigarettes that were sold at a self-reported retail price of R10 in 2017. Using a price elasticity of −0.6, the new quantity demanded is calculated using equation (2) as $Q_{2}=2.6\;billion\;cigarettes\;x\left[1+\left(-0.6\right)\left(\frac{23-10}{10+23}\right)\right]/\;\left[1-\left(-0.6\right)\left(\frac{23-10}{10\;+23}\right)\right]$ = 1.6 billion cigarettes. For cigarettes sold at R10 per pack, the percentage of cigarettes that would still have been bought, if the price had been R23, is 62% (1.6/2.6). We applied this principle to all prices below R23. We then calculated the new corresponding quantities for each price point. Finally, we summed all the new quantities. This yielded a new level of consumption of 7.4 billion cigarettes, down from 9.7 billion cigarettes. The proportion of cigarettes that would still have been bought if the price had been R23 is 0.76 (7.4/9.7). The other 24% of previously illicit sales are ‘lost’ (ie, not bought).

Since we only had granular price data for 2017, we used the same adjustment proportion (0.76) to account for the law of demand in all other years. For example, in 2022, the total lost excise revenue is calculated as 19.44 billion illicit cigarettes (= 0.97 billion packs), multiplied by the average excise tax for the calendar year 2022 (R19.56/pack), times 0.76, which equals R14.5 billion. The same is done for lost VAT revenue (for 2022: 0.97 billion packs x R4.13 VAT per pack x 0.76 = R3.1 billion). Applying the same proportion is a limitation of the paper as the proportion would differ from that in 2017 if the distribution of the prices of illicit sales for other years differs from the distribution of the prices of illicit sales in 2017. This assumption does not undermine the analysis in years when illicit trade was small since the proportion of 0.76 is applied to the number of illicit cigarettes. As a sensitivity analysis, we also used price elasticities of −0.4 (which yields an adjustment proportion of 0.83) and a price elasticity of −0.8 (adjustment proportion of 0.70).

### Final results and some sensitivity analyses

**Figure 3:**
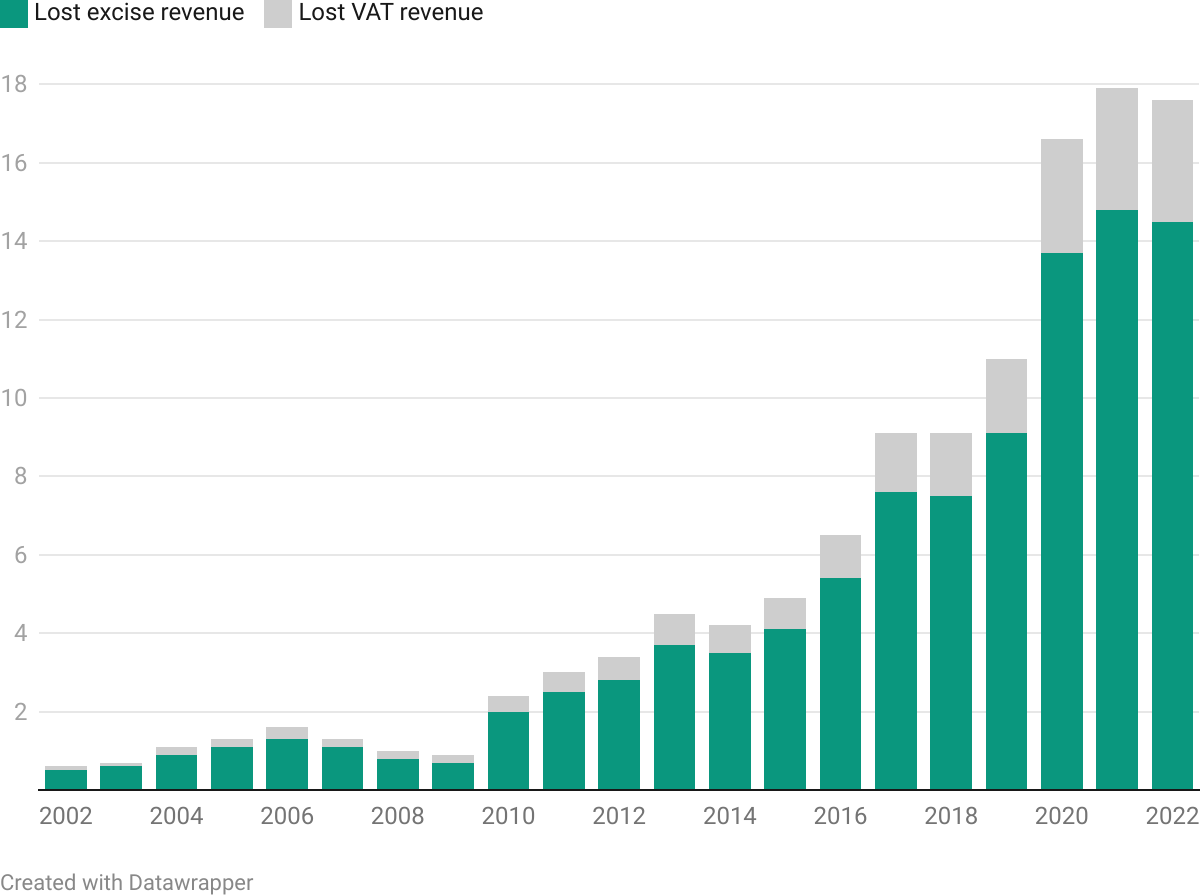
Lost excise and VAT revenue (billions of Rands, 2022 prices). VAT, value-added tax.

Our preferred specification has the following assumptions: price elasticity of −0.6, smoking prevalence of 23.4% for 2021 and 2022, and under-reporting of 5%. From 2002 to 2009, lost excise and VAT revenue accounted for less than R2 billion each year (2022 prices) (figure 3). From 2010, lost excise and VAT revenue steadily increased. From 2020, lost excise and VAT revenue exceeded R16 billion each year, peaking at R17.9 billion in 2021. The total amount of revenue lost between 2002 and 2022, expressed in 2022 prices, amounted to R118.8 billion (R98.3 billion excise tax and R20.5 billion VAT).

To account for the uncertainty around the various assumptions, we conducted several sensitivity analyses. We varied the following parameters: (1) price elasticity (−0.4, −0.6, and −0.8), (2) smoking prevalence for 2021 (20.0% and 23.4%), and the interpolated values for the period 2015 to 2020, and (3) under-reporting and over-reporting (5% and −5%), which results in 12 combinations. Over-reporting of −5% (the negative sign in effect means that we assume that cigarette consumption was *over*-reported) was chosen to account for the presence of roll-your-own (RYO) cigarettes. RYO cigarette prevalence has been increasing in South Africa,^32^ and it is possible that respondents included the use of RYO when they were asked about smoking. Combined data sets from 2010 to 2018 indicate that the percentage of smokers who were exclusive RYO smokers was 5.2%, dual smokers (manufactured cigarettes and RYO) was 21.1%, and exclusive manufactured cigarettes smokers was 73.7%.^33^ We consider 5% under-reporting (upper bound) as the baseline. Assuming that RYO cigarettes comprise 10% of the total number of cigarettes consumed, we apply an over-reporting adjustment of −5% (sensitivity analysis; lower bound) from 2010 to 2022 (a decrease of −5% before 2010 results in nonsensical negative illicit trade estimates). The lowest lost revenue estimate for the period 2002 to 2022 is R65.3 billion (in 2022 prices) (elasticity:−0.8, smoking prevalence in 2021: 20%, over-reporting of −5%, indicated in table 2 as −5%), while the highest is R129.7 billion (2022 prices) (elasticity:−0.4, smoking prevalence in 2021: 23.4%, under-reporting: 5%) (table 2).

**Table 2:** Sensitivity analyses

	Elasticity	Smoking prevalence estimate in 2021 (%)	Over-reporting and under-reporting (%)	Estimates of lost revenue(excise and VAT) (R billion) (2022 prices)
	(1)	(2)	(3)	(4)
Sensitivity analysis 1	−0.8	20.0	−5	65.3
Sensitivity analysis 2	−0.6	20.0	−5	71.2
Sensitivity analysis 3	−0.4	20.0	−5	77.7
Sensitivity analysis 4	−0.8	23.4	−5	81.0
Sensitivity analysis 5	−0.6	23.4	−5	88.3
Sensitivity analysis 6	−0.4	23.4	−5	96.4
Sensitivity analysis 7	−0.8	20.0	5	91.6
Sensitivity analysis 8	−0.6	20.0	5	99.9
Sensitivity analysis 9	−0.8	23.4	5	109.0
Sensitivity analysis 10	−0.4	20.0	5	109.0
**Main analysis**	−**0.6**	**23.4**	**5**	**118.8**
Sensitivity analysis 11	−0.4	23.4	5	129.7

Note that a negative sign in column 3 indicates over-reporting, while a positive sign indicates under-reporting.

## Discussion

The South African government lost R119 billion (2022 prices) in excise and VAT revenue from 2002 to 2022. A comprehensive sensitivity analysis indicates that the estimated lost revenue of R119 billion from 2002 to 2022 falls within the range of R65 billion to R130 billion (all 2022 prices). The majority of the lost revenue occurred in the period 2010 to 2022, when R110 billion (2022 prices) in excise and VAT revenue was lost. As a percentage of total revenue in 2022, tax-paid cigarettes contributed 0.6% to total revenue. If SARS had collected the additional R17.6 billion in lost revenue, an additional 1% would have been added to total government revenue.

In the context of a market with such a large illicit component, an increase in the excise tax becomes much less potent as a tobacco control tool. An increase in the excise tax will have an impact on the price of legal cigarettes, while the price of illicit cigarettes is unaffected (unless illicit cigarette manufacturers increase retail prices). Furthermore, the availability of illicit cigarettes makes it easy for smokers to switch to the illicit market. In the past few years, South Africa’s National Treasury has increased the excise tax by roughly the inflation rate. The National Treasury may be waiting for SARS to contain illicit trade, which, to date, SARS has not been able to do, despite some gains.

In 2022, SARS placed Gold Leaf Tobacco Corporation (GLTC) under curatorship on evidence that GLTC was involved in money laundering and tax evasion.^34^ While GLTC is probably the largest tobacco company evading taxes, research from 2020 indicates that illicit cigarette trade was ubiquitous during the COVID-19 cigarette sales ban,^7^ and illicit trade has not decreased substantially since the ban was lifted in August 2020.

In the 2023 budget speech, the Minister of Finance highlighted his concern about the illicit market. He mentioned several strategies that SARS has implemented to fight it. In the speech, the minister said: ‘On illicit trade, over the past three years, SARS has taken several steps to enhance its effectiveness in combating illicit trade, particularly in tobacco. To this end, SARS has completed 2316 seizures of cigarettes & tobacco products to the value of R598.8 million’.^35^ However, this number is only a small proportion of the illegal cigarettes in the market.

The illicit market threatens the 2018 Control of Tobacco Products and Electronic Delivery Systems Bill.^36^ Although this bill is more than 5 years old, it has not yet been enacted into law. The bill proposes to, inter alia, implement plain packaging, ban point-of-sale advertising, and make all indoor public places 100% smoke-free.^36^ If the bill is implemented, and the illicit cigarette market remains unchanged, the legislation may be undermined by the illicit market.

Reducing the availability of illicit cigarettes is likely to decrease smoking prevalence. A lower smoking prevalence, in the long run, reduces the substantial public health costs caused by smoking. A 2021 paper^3^ found that the economic cost of smoking in South Africa in 2016 was R42 billion (US$2.88 billion), of which R14.48 billion was for healthcare costs (hospitalisation and outpatient department visits).

We note several limitations to this study. First, for years with missing smoking prevalence data, we interpolated data for 2016–2020, and extrapolated data for 2022. For smoking intensity, we interpolated data for 2012–2019, and extrapolated data for 2021 and 2022. If our estimates for smoking prevalence and/or smoking intensity are higher (lower) than actual numbers, we over-estimate (under-estimate) the quantity of lost revenue. Second, the use of different data sets to estimate smoking behaviour reduces consistency. Data sets from different sources have distinct sampling designs and question wording, and might be subject to bias.^37^ Third, self-reported estimates from surveys suffer because of under-reported consumption.^38^,^40^ To address under-reporting, we scale up consumption by 5% (in the main analysis), as has been done in previous South African studies.^14 24^ In order to account for RYO cigarettes (which are subject to a much lower excise tax), we assumed that the self-reported number of manufactured cigarettes can be up to 10% lower than the (scaled-up) self-reported manufactured cigarette consumption. Fourth, the final computation of the size of the illicit market and the amount of lost tax revenue is an amalgam of many different estimates and assumptions, each of which is subject to a degree of uncertainty. To limit this uncertainty, we conducted several sensitivity analyses. Fifth, because we had granular price data for only 1 year (2017), we used the same adjustment proportion (0.76, using a price elasticity of −0.6) to account for the law of demand in all other years. If the distribution of prices of illicit sales for other years differs from the distribution of prices of illicit sales in 2017, the adjustment proportion would differ from that in 2017. For example, if there is a wide range of prices below the threshold price, the adjustment proportion would be larger than if prices are clustered just below the threshold price. Sixth, National Treasury’s cigarette excise revenue includes revenue from all Southern African Customs Union (SACU) countries (South Africa, Botswana, Lesotho, Eswatini, and Namibia). The number of smokers based on survey data used in our analysis applies only to South Africa. If it were possible to disaggregate the excise tax revenue data (thereby excluding excise revenue from Botswana, Lesotho, Eswatini, and Namibia), total legal sales in South Africa would be even less than calculated, and therefore illicit trade estimates would be even higher. However, since 94% of smokers in SACU reside in South Africa, the impact on the illicit trade is modest. Seventh, it is possible that smokers may have used the money they saved from buying cheaper untaxed cigarettes on other goods that could be subject to excise and VAT (eg, alcohol). To the extent that this happened, the tax revenue on other products may have increased. Eighth, three out of the five parameters used to determine the price threshold of R23 were based on information from the tobacco industry, which may be unreliable. However, these three parameters comprise about 30% of the threshold price, so even if there are errors, they should have only a modest impact on the magnitude of the threshold price.

## Conclusions

Cheap, tax-evaded cigarettes greatly reduce the effectiveness of tobacco taxes, and also reduce revenue. Tax-unpaid cigarettes represent a substantial loss to government. SARS should secure the cigarette supply chain to monitor cigarettes from the point of production to the point of sale. The Protocol to Eliminate Illicit Trade in Tobacco Products^41^ provides guidelines for reducing illicit trade. South Africa has not yet ratified the Protocol. The Protocol commits governments to take effective steps to reduce the illicit trade in tobacco products, such as allowing only licensed manufacturers to produce cigarettes and implementing a track-and-trace system. If SARS does not secure the supply chain, South Africa will continue to lose valuable revenue.

## Supplementary material

10.1136/bmjopen-2023-077855 online supplemental file 1

## Data Availability

Data are available in a public, open access repository.
