# Supplementary material for: Tax revenue lost due to illicit cigarettes in South Africa: 2002−2022
Source: BMJ Open. 2024 Mar 14;14(3):e077855. doi: 10.1136/bmjopen-2023-077855 (PMC10941134; doi:10.1136/bmjopen-2023-077855)

Supplemental material

Figure S1 Revenue from cigarette and cigarette tobacco (billions of Rands, 2022 prices)

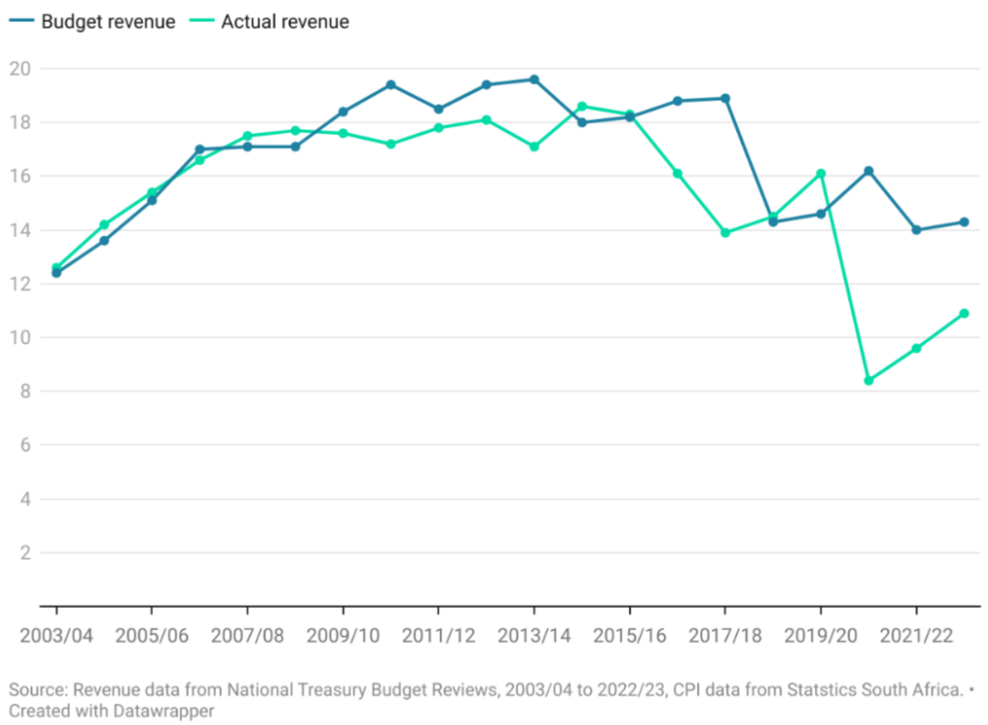

Figure S2 Cumulative percentage of cigarettes sold from R0 to R80 per pack in 2017 (nominal prices)

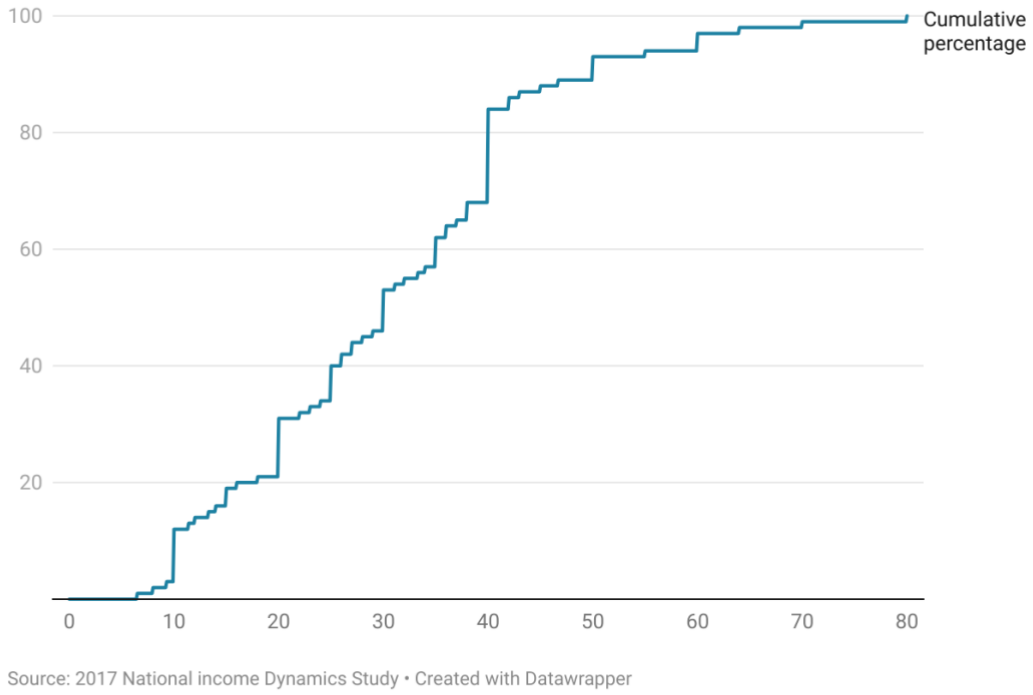

Supplement: online supplemental file 1 [file bmjopen-2023-077855supp001.pdf]
