# Supplementary material for: Tax revenue lost due to illicit cigarettes in South Africa: 2002−2022
Source: BMJ Open. 2024 Mar 14;14(3):e077855. doi: 10.1136/bmjopen-2023-077855 (PMC10941134; doi:10.1136/bmjopen-2023-077855)
Supplement: Supplementary file 2 [file bmjopen-2023-077855.reviewer_comments.pdf]

PEER REVIEW HISTORY

BMJ Open publishes all reviews undertaken for accepted manuscripts. Reviewers are asked to complete a checklist review form (**http://bmjopen.bmj.com/site/about/resources/checklist.pdf**) and are provided with free text boxes to elaborate on their assessment. These free text comments are reproduced below.

ARTICLE DETAILS

**TITLE (PROVISIONAL)**	An economic analysis of the tax revenue lost due to illicit cigarettes in South Africa: 2002 - 2022
**AUTHORS**	Vellios, Nicole; Walbeek, Corne

VERSION 1 – REVIEW

**REVIEWER**	Nkomo, Nomusa Yolanda<br>University of Zululand, Economics
**REVIEW RETURNED**	23-Aug-2023

**GENERAL COMMENTS**	The paper is exciting and addresses revenue issue, which is paramount to national development. The introduction is too long, and there is a need to limit it to one and a half page maximum. Some sentences make no meaning, and several incomplete sentences, such as "Momentum stagnated after 2011; However, from 2010 things changed dramatically". The conclusion omits important aspects of the study, and there is a need for a better explanation. Also, elaborate on " In addition, the public health costs are substantial." What are the recommendations? The authors should mention the study's limitations and recommend future research.

**REVIEWER**	Ayo-Yusuf, Olalekan<br>Sefako Makgatho Health Sciences University, Dean's office
**REVIEW RETURNED**	28-Aug-2023

**GENERAL COMMENTS**	This study sought to update the 2002 - 2017 time series estimates of the size of the illicit market in South Africa and extended this by estimating excise and VAT revenue lost by the government due to illicit market. The study innovatively applied arc formula of the price elasticity of demand and found a dramatic increase in illicit cigarette market peaking at 60% in 2021. While this may have important policy relevance, there are a number of assumptions made in the estimations that may limit its contribution to policy and/or to the existing body of knowledge on illicit market estimation. The authors may want to consider these issues as itemized below in improving the paper further;<br>Introduction<br>1. Page 5; lines 46-60: As it stands, the study is primarily motivated on the basis of loss of revenue and may leave the policy maker thinking this is the primary purpose of price increase through excise tax while the primary purpose was for improving health through reduction in cigarette consumption. Conceivably, there would be ‘loss of revenue’ if this policy instrument work well. I do not think there is enough said about this in the introduction. It cannot be assumed that the purpose of the tobacco excise tax is known to the reader. I suggest the authors consider strengthening the introduction (and perhaps in discussions too) by explicitly stating the

	purpose of the excise tax is not to generate revenue for the government and include the estimated cost of tobacco use in South Africa as estimated in 2016.<br>Methods<br>2. A major concern here remains that a number of unsupported assumptions are made to compute estimates of illicit consumption without sensitivity analysis on alternative scenarios.<br>Page 8; 26-33; Reading the literature from South Africa, it is conceivable that the number of cigarettes smoked per day may still be overestimated especially when one considers that the 23.9% prevalence of smoking as reported in GATS factsheet included non-daily smokers (~15% i.e. 3.4/23.9) and also includes roll-your-own (RYO) or hand-rolled cigarette smokers (see footnote 1 in the factsheet) which has been reported to be increasing in South Africa (see: Tob Induc Dis. 2023 Jul 17;21:94. doi: 10.18332/tid/168121). A previous study estimated that about 30% of smokers use RYO as at 2010 (see: BMC Public Health 2013 Jun 24;13:597. doi: 10.1186/1471-2458-13-597) and another study suggested 20% of RYO smokers as at 2018 did not smoke factory-manufactured cigarettes (i.e. exclusive users) (Tob Induc Dis. 2022; 20: 94. doi: 10.18332/tid/154798). These numbers are likely higher by 2021 considering that RYO was associated with low income status and possibly as a price minimization strategy. More so, that prices of factory-manufactured cigarettes (FMC) cigarettes reportedly increased by over 200% during the temporary ban of cigarettes sales in 2020. Even if these RYO smokers were also smoking FMC, they would probably be smoking significantly fewer FMC when compared to those who do not also smoke RYO. Similarly, non-daily smokers in South Africa have been previously reported to smoke significantly fewer cigarettes a day (5.4 vs. 9.8 or 45% less CPD than daily smokers) (See https://www.tandfonline.com/doi/abs/10.1080/20786204.2009.10873855). Moreover, it is conceivable that 'non-daily' smokers would not smoke for 365 days and I do not understand the argument that tries to justify why non-daily smokers' annual cigarette consumption would be computed based on 365days a year. A reasonable consumption of 20 days a month (i.e. 240days/year) as used as threshold for 'regular' smoking for young smokers might be reasonable to use for sensitivity analysis together with above mentioned parameters. I suggest the authors considers a sensitivity analysis that factors in these alternative scenarios. For example, if 30% of 10m smokers in 2021 were RYO smokers (3m) and 20% of these don't use FMC, then the base population would be 9,4m of whom 15% are non-daily smokers and another fraction concurrently using RYO. The later groups would be probably smoking about 5 sticks per day (except an alternative reference can be provided on CPD among daily and non-daily smokers and also among RYO+ FMC user vs. FMC only).<br>3. Page 10; line 14: Can it be assumed that SARS/government of South Africa did not received any of the amount (excise and, especially VAT/general sales tax) not spent on cigarettes through purchase of alternative goods with those 'savings' smokers made by shifting to cheaper cigarettes or quitting? Perhaps some comments on this in the discussion may help or even as an assumption/consideration in calculating 'lost venue'.<br><br>Discussion<br>4. The discussion and, in particular the limitations may need to be reworked based on some of the comments above. These are important to note so as not to make this an industry or even government instrument to oppose stronger tobacco control measure in relation to increasing excise tax, contrary to the authors' intent of motivating government action.

	5. Could the author comment on the fact that despite the estimated dramatic increase in the availability of cheap illicit cigarettes, smoking prevalence (even growth in count of smokers, except 2021) in the last 5 years of the studied period did not seem to have significantly change?<br>6. It should be noted that the 2021 estimate of 60% reported by the authors differs from industry-commissioned study’s actual overall (34% based on price threshold) or its media-reported exaggerated figure (70%). It is also pertinent to note that the estimates in this study also differed from that reported by Euromonitor for 2021 (37%) in its most recent report published in June 2023. Can the authors comment on these differences?

**REVIEWER**	Prieger, James E.<br>Pepperdine University, School of Public Policy
**REVIEW RETURNED**	19-Sep-2023

**GENERAL COMMENTS**	This article provides estimates of how much tax revenue the S. African government lost because of illicit trade in tobacco products (ITTP), specifically cigarettes, during 2002-2022. The methodology employed is the familiar gap analysis, wherein estimates of total consumption of cigarettes are compared with tax-paid sales, with the difference ascribed to ITTP. This methodology is well established in the tobacco control and health economics literature. While gap analysis is most often used to estimate the proportion of the total market that consists of ITTP, the present authors go further to compute estimates of the absolute amount of ITTP. Another extra step in the analysis is estimating what tax-paid sales would have been had illicit options been unavailable to consumers. The paper is interesting and the number of steps in the final computations is impressive. However, taking these extra steps results in the final computation for lost tax revenue being a complicated amalgam of many different estimates and assumptions, each of which is uncertain, but with no indication of the total level of uncertainty surrounding the final estimates.<br><br>Previous reviewers (in particular, reviewer #3) have already enumerated the many assumptions, extrapolations, arbitrary smoothing procedures, noncomparable surveys, etc. that are used in the calculations. None of the individual choices the researchers made in regard to these appears to be incorrect, necessarily. My point here is a different one. The final numerical conclusions rest upon an intricate set of estimates and calculations. However, there are no confidence intervals, credibility intervals, or sensitivity analyses performed. To take a few examples: the elasticity figure of 0.6 is drawn from the literature to represent a developing country's price sensitivy of demand for cigarettes. Of course, any study so relied upon reported a point estimate AND a confidence interval. Errors and uncertainty in the point estimate will be promulgated throughout the rest of the computations, results in a _range_ of final estimates for the tax lost. If the elasticity were half as large, what would the implications be? What if it were twice as large? We have no way of knowing. The same applies to the almost-but-not-quite arbitrary figure of 5% chosen for the underreporting of consumption from the tobacco use surveys. It may be the best guess, or at least a good one, but what if it were half or twice as large?<br><br>Point estimates without uncertainty intervals or sensitivity analyses of some sort lend a false sense of precision to empirical results, especially for policy-relevant work where non-statisticians will

	compose an important part of the readership. I strongly suggest that sensitivity analyses be conducted (and not just by varying one parameter at a time, since joint errors in assumption could greatly compound the final difference in estimates, given that the ultimate figures are not purely linear combinations of the various assumed parameters. Certainly for a journal at the level of BMJ Open such analysis would seem to be warranted, especially since otherwise the paper is largely an updating of an earlier article undertaking a substantially similar exercise for the same country in any early period.<br><br>The above is my major comment on this research. Before passing to minor comments, I mention two other points that seem important to me.<br><br>First, since the NIDS 2017 survey data supply a key parameter for the analysis (the markdown figure for actual illicit quantity to counterfactual licit quantity purchased by consumers who otherwise bought illicit) it would seem important to see how they square with your other data and assumptions. That is, from the NIDS one could presumably compute an independent estimate of the prevalence of ITTP (at least as a fraction of the market if not the total amount, but probably even the latter). How do such estimates compare with those from your gap analysis? If they are way off, then it would possibly cast some doubt on the "0.76" parameter derived from the NIDS used later in the computations, since the dataset wouldn't seem to lead to externally valid results. On the other hand, concordance between NIDS estimates of ITTP and you gap method would bolster confidence in both.<br><br>Second, neither of the conclusions mentioned in the abstract, strictly speaking, follow from the statistical work in the paper. The first "conclusion" is that "The illicit cigarette market in South Africa is one of the largest in the world." That may be true, but since there are no comparisons in the paper the reader cannot draw this conclusion. The other conclusion is a call for track and trace (T&T). Again, this may be a sensible policy step, but it does not _follow_ from the research in this paper. Given that this is not an editorial, and that BMJ is a scientific journal, I would suggest either limiting the conclusions to the actual scientific results in the paper or expanding on why T&T would solve the problem (although that might be another paper). Right now that conclusion has a "tacked on" feel to it.<br><br>I note a few other considerations for the authors below.<br><br>1. How does the assumed price elasticity of 0.6 square with the statement that the sales ban during COVID reduced tax revenue but "was unaccompanied by a decrease in smoking prevalence"? When cigarette price elasticity is broken down, usually about half comes from the extensive margin (prevalence) and the other half from the intensive margin (cigarettes per day). So why would we expect that an off-the-shelf figure of 0.6 (which does not appear to be based on econometric estimates from S.A. in particular) is an appropriate one? (Again, it may be the best guess, but the uncertainties surround it cry out for sensitivity analysis).<br><br>2. Along with a previous reviewer I am puzzled why the AMPS data were smoothed with a moving-average procedure. I understand the thought behind it, which is that the estimates year

	to year are noisy. But wouldn't it be better to treat the noise honestly, promulgating the estimates' standard errors all the way through to the final estimate, which could then (at least) account for that source of estimation error?<br><br>3. Partially regarding point 1 above, later in the paper we read that "the 20 week sales ban ... temporarily reduced the number of smokers by approximately 10%" But earlier the paper seemed to say that the ban had no effect on prevalence. In the earlier instance, was it meant that there was no _lasting_ effect? Perhaps this can be clarified in the text.<br><br>4. Equation 1 is incorrect. The division bar should actually be a multiplication symbol. Nevertheless, Eqn. 2 is correct.<br><br>5. It is too strong a statement to claim that "This assumption [that illicit quantity can be multiplied by 0.76 to find the counterfactual Law-of-Demand adjusted quantity in the absence of ITTP] does not undermine the analysis in any way in years when illicit trade was small since the proportion of 0.76 is applied to the number of illicit cigarettes." Clearly "in any way" is literally false. What you mean to say is that the errors so introduced in such years would not affect your final total lost tax estimates _much_.<br><br>6. It is stated that:<br>"...SARS has completed 2,316 seizures of cigarettes & tobacco products to the value of R598.8<br>million. However, this number is only a small proportion of the illegal cigarettes in the<br>market. We estimate that SARS lost around R15 billion in excise revenue in 2022. The R598.8 million is only about 4% of the actual lost revenue"<br>This appears to be comparing apples to oranges. Isn't SARS stating that the _market value_ (tax inclusive but not _just_ the taxes) is R598? Whereas your figure is just for taxes.<br><br>7. The sentence "Any sale at a retail price of less than R23.00 indicates that taxes were not (fully) paid" is repeated on p.11. This appears to be inadvertent.<br><br>8. Finally, I note that the list of limitations in publication #9, with substantially similar methodology and some of the same authors, appears to be more complete than the limitations stated for the present article.

VERSION 1 – AUTHOR RESPONSE

Reviewer: 1 (Dr. Nomusa Yolanda Nkomo, University of Zululand)

Comments to the Author:

The paper is exciting and addresses revenue issue, which is paramount to national development. The introduction is too long, and there is a need to limit it to one and a half page maximum. Some sentences make no meaning, and several incomplete sentences, such as "Momentum stagnated after 2011; However, from 2010 things changed dramatically". The conclusion omits important aspects of

the study, and there is a need for a better explanation. Also, elaborate on " In addition, the public health costs are substantial." What are the recommendations? The authors should mention the study's limitations and recommend future research.

Response: We have shortened the introduction substantially.

We considered adding information after the ‘In addition, the public health costs are substantial’ sentence. A 2021 paper[4] found that the economic cost of smoking in South Africa in 2016 was R42 billion (US$2.88 billion), of which R14.48 billion was for healthcare costs (hospitalization and outpatient department visits). Given that the sentence is in the conclusion, and the last sentence, we decided to rather delete it.

Regarding recommendations, in the conclusions we say “SARS should secure the cigarette supply chain to monitor cigarettes from the point of production to the point of sale. The Protocol to Eliminate Illicit Trade in Tobacco Products[5] provides guidelines for reducing illicit trade. South Africa has not yet ratified the Protocol. The Protocol commits governments to take effective steps to reduce the illicit trade in tobacco products, such as allowing only licensed manufacturers to produce cigarettes and implementing a track-and-trace system. If SARS does not secure the supply chain, South Africa will continue to lose valuable revenue.”

The limitation section now reads as follows:
“We note several limitations to this study. First, for years with missing smoking prevalence, we interpolated data for 2016 □ 2020, and extrapolated data for 2022. For smoking intensity, we interpolated data for 2012 □ 2019, and extrapolated data and 2021 and 2022. If our estimates for any of these two variables are higher (lower) than actual numbers, we over-estimate (under-estimate) the quantity of lost revenue. Second, the use of different datasets to estimate smoking behaviour reduces consistency. Datasets from different sources have distinct sampling designs and question wording. Third, self-reported estimates from surveys suffer because of under-reported consumption.[6-8] To address under-reporting we scale up consumption by 5% (in the main analysis) and 10% (in the sensitivity analysis). Fourth, the final computation for lost tax revenue is an amalgam of many different estimates and assumptions, each of which is uncertain. To limit this uncertainty, we conducted several sensitivity analyses. Fifth, because we had granular price data for only one year (2017), we used the same adjustment proportion (0.76, using a price elasticity of □0.6) to account for the law of demand in all other years. If the distribution of prices of illicit sales for other years differs from the distribution of prices of illicit sales in 2017, the adjustment proportion would differ from that in 2017. For example, if there is a wide range of prices below the threshold price, the adjustment proportion would be larger than if prices are clustered just below the threshold price. Sixth, National Treasury’s cigarette excise revenue includes revenue from all Southern African Customs Union (SACU) countries (South Africa, Botswana, Lesotho, Eswatini, and Namibia). The number of smokers based on survey data used in our analysis applies only to South Africa. If it were possible to disaggregate the excise tax revenue data (thereby excluding excise revenue from Botswana, Lesotho, Eswatini, and Namibia), total legal sales in South Africa would be even less than calculated (so illicit trade estimates would be even higher). However, since 94% of smokers in SACU reside in South Africa the impact on the illicit trade is modest. Seventh, it is possible that smokers may have used the money they saved from buying cheaper untaxed cigarettes on other goods that could be subject to excise and VAT (e.g., alcohol). To the extent that this happened, the tax revenue on other products may have increased.”

We can’t think of any avenues for future research. We have extended the gap analysis to a new area of research so perhaps our methods could be replicated in other countries.

Reviewer: 2 (Prof. Olalekan Ayo-Yusuf, Sefako Makgatho Health Sciences University, Harvard School of Public Health)

Comments to the Author:

This study sought to update the 2002 - 2017 time series estimates of the size of the illicit market in South Africa and extended this by estimating excise and VAT revenue lost by the government due to illicit market. The study innovatively applied arc formula of the price elasticity of demand and found a dramatic increase in illicit cigarette market peaking at 60% in 2021. While this may have important policy relevance, there are a number of assumptions made in the estimations that may limit its contribution to policy and/or to the existing body of knowledge on illicit market estimation. The authors may want to consider these issues as itemized below in improving the paper further.
Response: Thank you for taking the time to review the paper.

Comment 1: Page 5; lines 46-60: As it stands, the study is primarily motivated on the basis of loss of revenue and may leave the policy maker thinking this is the primary purpose of price increase through excise tax while the primary purpose was for improving health through reduction in cigarette consumption. Conceivably, there would be 'loss of revenue' if this policy instrument work well. I do not think there is enough said about this in the introduction. It cannot be assumed that the purpose of the tobacco excise tax is known to the reader. I suggest the authors consider strengthening the introduction (and perhaps in discussions too) by explicitly stating the purpose of the excise tax is not to generate revenue for the government and include the estimated cost of tobacco use in South Africa as estimated in 2016.

Response to comment 1: Thank you for pointing this out. We have added the below paragraph to the paper, which is now the opening paragraph of the intro:
"The purpose of cigarette excise taxes is to reduce smoking prevalence. Increasing excise taxes is the most effective tobacco control policy to reduce smoking prevalence 9 10 Lowering smoking prevalence decreases the substantial burden of disease caused by smoking, which was estimated to be around 25 700 deaths among South Africans aged 35−74 in 2016 4."

Methods
Comment 2: A major concern here remains that a number of unsupported assumptions are made to compute estimates of illicit consumption without sensitivity analysis on alternative scenarios.
Page 8; 26-33; Reading the literature from South Africa, it is conceivable that the number of cigarettes smoked per day may still be overestimated especially when one considers that the 23.9% prevalence of smoking as reported in GATS factsheet included non-daily smokers (~15% i.e. 3.4/23.9) and also includes roll-your-own (RYO) or hand-rolled cigarette smokers (see footnote 1 in the factsheet) which has been reported to be increasing in South Africa (see: Tob Induc Dis. 2023 Jul 17;21:94. doi: 10.18332/tid/168121). A previous study estimated that about 30% of smokers use RYO as at 2010 (see: BMC Public Health 2013 Jun 24;13:597. doi: 10.1186/1471-2458-13-597) and another study suggested 20% of RYO smokers as at 2018 did not smoke factory-manufactured cigarettes (i.e. exclusive users) (Tob Induc Dis. 2022; 20: 94. doi: 10.18332/tid/154798). These numbers are likely higher by 2021 considering that RYO was associated with low income status and possibly as a price minimization strategy. More so, that prices of factory-manufactured cigarettes (FMC) cigarettes reportedly increased by over 200% during the temporary ban of cigarettes sales in 2020. Even if these RYO smokers were also smoking FMC, they would probably be smoking significantly fewer FMC when compared to those who do not also smoke RYO. Similarly, non-daily smokers in South Africa have been previously reported to smoke significantly fewer cigarettes a day (5.4 vs. 9.8 or 45% less CPD than daily smokers)
(See https://www.tandfonline.com/doi/abs/10.1080/20786204.2009.10873855). Moreover, it is conceivable that 'non-daily' smokers would not smoke for 365 days and I do not understand the

argument that tries to justify why non-daily smokers' annual cigarette consumption would be computed based on 365days a year. A reasonable consumption of 20 days a month (i.e. 240days/year) as used as threshold for 'regular' smoking for young smokers might be reasonable to use for sensitivity analysis together with above mentioned parameters. I suggest the authors considers a sensitivity analysis that factors in these alternative scenarios. For example, if 30% of 10m smokers in 2021 were RYO smokers (3m) and 20% of these don't use FMC, then the base population would be 9,4m of whom 15% are non-daily smokers and another fraction concurrently using RYO. The later groups would be probably smoking about 5 sticks per day (except an alternative reference can be provided on CPD among daily and non-daily smokers and also among RYO+ FMC user vs. FMC only).

Response to comment 2:
According to the reviewer's article (Tob Induc Dis. 2023 Jul 17;21:94. doi: 10.18332/tid/168121), smoking prevalence has been on the rise since 2007. The paper states that current cigarette increased from 19.3% in 2007 to 20.4% in 2018 (with a strange decrease to 16.1% in 2010). We agree with the overall trend that smoking prevalence has been increasing, which confirms that we should be using the higher smoking prevalence from GATS 2021 (23.9% instead of 20.5%). The reviewer's paper also shows RYO prevalence increasing from 4.2% in 2007 to 7.9% in 2018. We observe that these percentages are based on small sample sizes.

To check whether or not the estimates for GATS 2021 are high because of RYO, we contacted Dr Catherine Edge from the South African Medical Research Council, asking her to parse out RYO and kreteks from the current cigarette smoking prevalence of 23.9%. Despite many requests, the GATS 2021 dataset is not publicly available. This significantly reduces our ability to conduct our own data analysis, and we are at the mercy of researchers who were involved with data collection and whom have access to the data. Dr Egbe sent us the table below, showing that manufactured cigarette smoking prevalence is 23.4%. We have therefore adjusted our number using this lower prevalence. We also conducted a sensitivity analysis where we also use a manufactured cigarette smoking prevalence of 20%. We also note that according to GATS, RYO prevalence is 2.8%, which is significantly lower than the 2018 estimate of 7.9% in the Tob Induc Dis. paper.

The reviewer mentions the increased use of RYO citing papers that are based on the South African Social Attitudes Surveys (SASAS). The finding that the prevalence of RYO smoking has nearly doubled between 2007 and 2018 seems peculiar. Between 2007 and 2018 the market for illicit cigarettes has increased from negligible levels to about 30□35%.1 The large increase in the illicit cigarette market means that cheap cigarettes are available to people who previously could not afford fully-taxed cigarettes, and were thus forced to purchase RYO cigarettes. RYO tobacco is generally consumed by poor consumers because they cannot afford manufactured cigarettes. It then is a strange finding that, in an environment where more desirable alternatives (i.e., cheap illicit manufactured cigarettes) are much more freely available, that there would be a very substantial increase in the prevalence of RYO cigarettes sold. We believe that the findings from the 2021 Global Adult Tobacco Study, showing a prevalence of RYO of around 2.8%, is probably more accurate. It is interesting to note also that the prevalence of any cigarette use only increases by 0.5% percentage points (from 23.4% to 23.9%) when RYO (and kretek) is included, indicating that dual use of manufactured and RYO cigarettes is substantial.

The text in the data section now reads as follows:
"Although the granular data have not yet been released, a factsheet indicates a current cigarette smoking prevalence (which includes manufactured cigarettes, hand-rolled cigarettes, and kreteks) of 23.9% among those aged 15+.11 The lead researcher of the GATS 2021 data collection, indicated

that current manufactured cigarette smoking prevalence was 23.4%. As part of a sensitivity analysis, we also used daily manufactured cigarette prevalence of 20% in 2021.”

We have also included the below in the results section:
“To account for the uncertainty around the various assumptions, we conducted several sensitivity analyses. We varied the following parameters: (1) price elasticity (□□□□, □□□6, and □□□8), (2) smoking prevalence for 2021 (20.0% and 23.4%), and the interpolated values for the period 2016 to 2020, and (3) under-reporting (5% and 10%), which results in 12 combinations. The lowest lost revenue estimate for the period 2002 to 2022 is R91.7 billion (in 2022 prices) (elasticity:□□□8, smoking prevalence in 2021: 20%, under-reporting: 5%), while the highest is R157.0 billion (2022 prices) (elasticity:□□□4, smoking prevalence in 2021: 23.4%, under-reporting: 10%).”

Table 2 Sensitivity analyses

	Elasticity	Smoking prevalence in 2021	Under-reporting	Estimates of lost revenue (excise and VAT) (2022 prices)
Sensitivity analysis 1	□□□□	20.0%	5%	91.7
Sensitivity analysis 2	□□□□	20.0%	5%	99.9
Sensitivity analysis 3	□□□□	23.4%	5%	109.0
Sensitivity analysis 4	□□□□	20.0%	5%	109.1
Sensitivity analysis 5	□□□□	20.0%	10%	113.6
Main analysis	□□□□	23.4%	5%	118.8
Sensitivity analysis 6	□□□□	20.0%	10%	123.8
Sensitivity analysis 7	□□□□	23.4%	5%	129.7
Sensitivity analysis 8	□□□□	23.4%	10%	131.9
Sensitivity analysis 9	□□□□	20.0%	10%	135.2
Sensitivity analysis 10	□□□□	23.4%	10%	143.8
Sensitivity analysis 11	□□□□	23.4%	10%	157.0

We have also substantially reworked the limitations section. See response to comment 4 below.

Comment 3: Page 10; line 14: Can it be assumed that SARS/government of South Africa did not received any of the amount (excise and, especially VAT/general sales tax) not spent on cigarettes through purchase of alternative goods with those ‘savings’ smokers made by shifting to cheaper cigarettes or quitting? Perhaps some comments on this in the discussion may help or even as an assumption/consideration in calculating ‘lost venue’.

Response to comment 3:
This is an insightful comment. It seems possible, but without using something like a social accounting matrix, it would be impossible to get an indication of this diverted spending. We have added this to the limitations section: “Seventh, it is possible that smokers may have used the money they saved from buying cheaper untaxed cigarettes on other goods that could be subject to excise and VAT (e.g., alcohol). To the extent that this happened, the tax revenue on other products may have increased.”

Discussion
Comment 4: The discussion and, in particular the limitations may need to be reworked based on some of the comments above. These are important to note so as not to make this an industry or even government instrument to oppose stronger tobacco control measure in relation to increasing excise tax, contrary to the authors’ intent of motivating government action.

Response to comment 4: We have substantially reworked the limitations section.
The limitation section now reads as follows:
“We note several limitations to this study. First, for years with missing smoking prevalence, we interpolated data for 2016 □ 2020, and extrapolated data for 2022. For smoking intensity, we

interpolated data for 2012 – 2019, and extrapolated data and 2021 and 2022. If our estimates for any of these two variables are higher (lower) than actual numbers, we over-estimate (under-estimate) the quantity of lost revenue. Second, the use of different datasets to estimate smoking behaviour reduces consistency. Datasets from different sources have distinct sampling designs and question wording. Third, self-reported estimates from surveys suffer because of under-reported consumption.6-8 To address under-reporting we scale up consumption by 5% (in the main analysis) and 10% (in the sensitivity analysis). Fourth, the final computation for lost tax revenue is an amalgam of many different estimates and assumptions, each of which is uncertain. To limit this uncertainty, we conducted several sensitivity analyses. Fifth, because we had granular price data for only one year (2017), we used the same adjustment proportion (0.76, using a price elasticity of −0.6) to account for the law of demand in all other years. If the distribution of prices of illicit sales for other years differs from the distribution of prices of illicit sales in 2017, the adjustment proportion would differ from that in 2017. For example, if there is a wide range of prices below the threshold price, the adjustment proportion would be larger than if prices are clustered just below the threshold price. Sixth, National Treasury's cigarette excise revenue includes revenue from all Southern African Customs Union (SACU) countries (South Africa, Botswana, Lesotho, Eswatini, and Namibia). The number of smokers based on survey data used in our analysis applies only to South Africa. If it were possible to disaggregate the excise tax revenue data (thereby excluding excise revenue from Botswana, Lesotho, Eswatini, and Namibia), total legal sales in South Africa would be even less than calculated (so illicit trade estimates would be even higher). However, since 94% of smokers in SACU reside in South Africa the impact on the illicit trade is modest. Seventh, it is possible that smokers may have used the money they saved from buying cheaper untaxed cigarettes on other goods that could be subject to excise and VAT (e.g., alcohol). To the extent that this happened, the tax revenue on other products may have increased."

Comment 5: Could the author comment on the fact that despite the estimated dramatic increase in the availability of cheap illicit cigarettes, smoking prevalence (even growth in count of smokers, except 2021) in the last 5 years of the studied period did not seem to have significantly change?

Response to comment 5:
We spent a lot of time thinking about this question. We agree with the reviewer that the presence of large numbers of illicit cigarettes would have likely increased the number of smokers. We estimate that smoking prevalence increased from 22.5% in 2018 to 23.4% in 2022. Although the percentage is small, it translates to a substantial increase in the absolute number of smokers. In 2018, we estimated that there were 9.2 million smokers, increasing to 10.0 million smokers in 2022.

While we included a scenario where smoking prevalence decreased to 20% in 2022 (with a small dip in 2020 to account for the sales ban), based on earlier comments of the reviewer, we believe that this 20% estimate for 2022 would be a lower limit prevalence percentage. Our understanding of economics and the vast literature on the demand for cigarettes suggest that smoking prevalence would have increased in a period where cheap illicit cigarettes became substantially more prevalent.

Comment 6: It should be noted that the 2021 estimate of 60% reported by the authors differs from industry-commissioned study's actual overall (34% based on price threshold) or its media-reported exaggerated figure (70%). It is also pertinent to note that the estimates in this study also differed from that reported by Euromonitor for 2021 (37%) in its most recent report published in June 2023. Can the authors comment on these differences?

Response to comment 6: A thorough analysis of the industry's numbers has been done (by the first author). See here: https://www.econ3x3.org/article/how-big-illicit-cigarette-market-south-africa. We could cite this but then it may seem like we are self-citing extensively. While our estimate of the illicit

market might be larger than the industry’s estimate (which is a rare occurrence), we do not want to give credibility to the industry’s estimates in an academic paper.

While Euromonitor International data is useful for some things, it is not useful for illicit trade estimates. See here: https://pubmed.ncbi.nlm.nih.gov/23792325/. We don’t feel it deserves any attention in our paper and we excluded it on purpose.

Reviewer: 3
Dr. James E. Prieger, Pepperdine University

Comments to the Author:

This article provides estimates of how much tax revenue the S. African government lost because of illicit trade in tobacco products (ITTP), specifically cigarettes, during 2002-2022. The methodology employed is the familiar gap analysis, wherein estimates of total consumption of cigarettes are compared with tax-paid sales, with the difference ascribed to ITTP. This methodology is well established in the tobacco control and health economics literature. While gap analysis is most often used to estimate the proportion of the total market that consists of ITTP, the present authors go further to compute estimates of the absolute amount of ITTP. Another extra step in the analysis is estimating what tax-paid sales would have been had illicit options been unavailable to consumers. The paper is interesting and the number of steps in the final computations is impressive. However, taking these extra steps results in the final computation for lost tax revenue being a complicated amalgam of many different estimates and assumptions, each of which is uncertain, but with no indication of the total level of uncertainty surrounding the final estimates.

Previous reviewers (in particular, reviewer #3) have already enumerated the many assumptions, extrapolations, arbitrary smoothing procedures, noncomparable surveys, etc. that are used in the calculations. None of the individual choices the researchers made in regard to these appears to be incorrect, necessarily. My point here is a different one. The final numerical conclusions rest upon an intricate set of estimates and calculations. However, there are no confidence intervals, credibility intervals, or sensitivity analyses performed. To take a few examples: the elasticity figure of 0.6 is drawn from the literature to represent a developing country's price sensitivity of demand for cigarettes. Of course, any study so relied upon reported a point estimate AND a confidence interval. Errors and uncertainty in the point estimate will be promulgated throughout the rest of the computations, results in a _range_ of final estimates for the tax lost. If the elasticity were half as large, what would the implications be? What if it were twice as large? We have no way of knowing. The same applies to the almost-but-not-quite arbitrary figure of 5% chosen for the underreporting of consumption from the tobacco use surveys. It may be the best guess, or at least a good one, but what if it were half or twice as large?

Point estimates without uncertainty intervals or sensitivity analyses of some sort lend a false sense of precision to empirical results, especially for policy-relevant work where non-statisticians will compose an important part of the readership. I strongly suggest that sensitivity analyses be conducted (and not just by varying one parameter at a time, since joint errors in assumption could greatly compound the final difference in estimates, given that the ultimate figures are not purely linear combinations of the various assumed parameters. Certainly for a journal at the level of BMJ Open such analysis would seem to be warranted, especially since otherwise the paper is largely an updating of an earlier article undertaking a substantially similar exercise for the same country in any early period.

Response: This is an excellent point. We have done additional calculations and we have added this to the paper:

“To account for the uncertainty around the various assumptions, we conducted several sensitivity analyses. We varied the following parameters: (1) price elasticity (□□□□, □□□6, and □□□8), (2) smoking prevalence for 2021 (20.0% and 23.4%), and (3) under-reporting (5% and 10%), which results in 12 permutations. The lowest estimated lost revenue estimate for the period 2002 to 2022 is R91.7 billion (2022 prices) (elasticity:□□□8, smoking prevalence: 20%, under-reporting: 5%), while the highest is R157.0 billion (2022 prices) (elasticity:□□□4, smoking prevalence: 23.4%, under-reporting: 10%) (Table 2).”

Table 2 Sensitivity analyses

	Elasticity	Smoking prevalence in 2021	Under-reporting	Estimates of lost revenue (excise and VAT) (2022 prices)
Sensitivity analysis 1	□□□□	20.0%	5%	91.7
Sensitivity analysis 2	□□□□	20.0%	5%	99.9
Sensitivity analysis 3	□□□□	23.4%	5%	109.0
Sensitivity analysis 4	□□□□	20.0%	5%	109.1
Sensitivity analysis 5	□□□□	20.0%	10%	113.6
Main analysis	□□□□	23.4%	5%	118.8
Sensitivity analysis 6	□□□□	20.0%	10%	123.8
Sensitivity analysis 7	□□□□	23.4%	5%	129.7
Sensitivity analysis 8	□□□□	23.4%	10%	131.9
Sensitivity analysis 9	□□□□	20.0%	10%	135.2
Sensitivity analysis 10	□□□□	23.4%	10%	143.8
Sensitivity analysis 11	□□□□	23.4%	10%	157.0

Comment: The above is my major comment on this research. Before passing to minor comments, I mention two other points that seem important to me.

First, since the NIDS 2017 survey data supply a key parameter for the analysis (the markdown figure for actual illicit quantity to counterfactual licit quantity purchased by consumers who otherwise bought illicit) it would seem important to see how they square with your other data and assumptions. That is, from the NIDS one could presumably compute an independent estimate of the prevalence of ITTP (at least as a fraction of the market if not the total amount, but probably even the latter). How do such estimates compare with those from your gap analysis? If they are way off, then it would possibly cast some doubt on the "0.76" parameter derived from the NIDS used later in the computations, since the dataset wouldn't seem to lead to externally valid results. On the other hand, concordance between NIDS estimates of ITTP and you gap method would bolster confidence in both.

Response: We compared AMPS and NIDS data in the original gap analysis paper,1 therefore we did not want to repeat the comparison in the current paper. Given that the trends in AMPS and NIDS are similar, we decided to use MAPS, which goes further back in time.

Comment: Second, neither of the conclusions mentioned in the abstract, strictly speaking, follow from the statistical work in the paper. The first "conclusion" is that "The illicit cigarette market in South Africa is one of the largest in the world." That may be true, but since there are no comparisons in the paper the reader cannot draw this conclusion. The other conclusion is a call for track and trace (T&T). Again, this may be a sensible policy step, but it does not _follow_ from the research in this paper. Given that this is not an editorial, and that BMJ is a scientific journal, I would suggest either limiting the conclusions to the actual scientific results in the paper or expanding on why T&T would solve the problem (although that might be another paper). Right now that conclusion has a "tacked on" feel to it.

Response: We agree with the author on the first point. We have deleted the sentence that says that SA has one of the largest rates of illicit trade. On the second point, we believe that suggesting a track

and trace system is a relevant conclusion of the paper and as such should remain in the paper. Regarding the tacked on feel of the conclusion, we have reworking the conclusion:

Comment: I note a few other considerations for the authors below.

Comment 1: How does the assumed price elasticity of 0.6 square with the statement that the sales ban during COVID reduced tax revenue but "was unaccompanied by a decrease in smoking prevalence"? When cigarette price elasticity is broken down, usually about half comes from the extensive margin (prevalence) and the other half from the intensive margin (cigarettes per day). So why would we expect that an off-the-shelf figure of 0.6 (which does not appear to be based on econometric estimates from S.A. in particular) is an appropriate one? (Again, it may be the best guess, but the uncertainties surround it cry out for sensitivity analysis).

Response: We use the phrase “unaccompanied by a decreased in smoking prevalence” in the following sentence: “The decrease in excise revenue, unaccompanied by a decrease in smoking prevalence, reflects the burgeoning illicit market.” This is to make it clear that excise revenue is not decreasing because smoking prevalence is decreasing.

In the text, we say “We used a price elasticity of □0.6, as this is frequently found in developing countries,12 including South Africa.13 14” We’ve also added “As a sensitivity analysis, we also use price elasticities of □0.4 and □0.8”.

Regarding the issue of the very low consumption response to large price increase during the covid-19 sales ban, this is discussed in detail in Van der Zee et al 2022.15 In that paper, the authors argued that all smokers knew that the sales ban was temporary and that it was a question of time when the ban would be lifted. From that study it was clear that most smokers decided to grin and bear the temporary price hikes. As such, we did not see, and did not expect, a substantial decrease in cigarette consumption, despite the very substantial temporary increase in the price. We have used two additional price elasticity estimates (□0.4 and □0.8) in the sensitivity analyses.

Comment 2: Along with a previous reviewer I am puzzled why the AMPS data were smoothed with a moving-average procedure. I understand the thought behind it, which is that the estimates year to year are noisy. But wouldn't it be better to treat the noise honestly, promulgating the estimates' standard errors all the way through to the final estimate, which could then (at least) account for that source of estimation error?

Response: We conducted the analysis with no data smoothing. The overall lost revenue increases from R118.8 billion to R119.0 billion.

Comment 3: Partially regarding point 1 above, later in the paper we read that "the 20 week sales ban ... temporarily reduced the number of smokers by approximately 10%" But earlier the paper seemed to say that the ban had no effect on prevalence. In the earlier instance, was it meant that there was no _lasting_ effect? Perhaps this can be clarified in the text.

Response: We are unable to find the first instance where we imply that the ban had no effect on prevalence. We searched for “ban” but none of the sentences with the word “ban” comment on its effect on prevalence.

Comment 4: Equation 1 is incorrect. The division bar should actually be a multiplication symbol. Nevertheless, Eqn. 2 is correct.

Response: thanks for picking this up. We have made the correction.

Comment 5: It is too strong a statement to claim that "This assumption [that illicit quantity can be multiplied by 0.76 to find the counterfactual Law-of-Demand adjusted quantity in the absence of ITTP] does not undermine the analysis in any way in years when illicit trade was small since the proportion of 0.76 is applied to the number of illicit cigarettes." Clearly "in any way" is literally false. What you mean to say is that the errors so introduced in such years would not affect your final total lost tax estimates _much_.

Response: Thank you for picking this up. We have deleted “in any way”.

Comment 6: It is stated that: "...SARS has completed 2,316 seizures of cigarettes & tobacco products to the value of R598.8 million. However, this number is only a small proportion of the illegal cigarettes in themarket. We estimate that SARS lost around R15 billion in excise revenue in 2022. The R598.8 million is only about 4% of the actual lost revenue". This appears to be comparing apples to oranges. Isn't SARS stating that the _market value_ (tax inclusive but not _just_ the taxes) is R598? Whereas your figure is just for taxes.

Response: True. We have deleted the last two sentences “We estimate that SARS lost around R15 billion in excise revenue in 2022. The R598.8 million is only about 4% of the actual lost revenue”, which resolves the issue.

Comment 7: The sentence "Any sale at a retail price of less than R23.00 indicates that taxes were not (fully) paid" is repeated on p.11. This appears to be inadvertent.

Response: Yes, this was a mistake. Thanks for picking it up. We have delete the repeated sentence.

Comment 8: Finally, I note that the list of limitations in publication #9, with substantially similar methodology and some of the same authors, appears to be more complete than the limitations stated for the present article.

Response:
We have expanded the limitations substantially. The limitation section now reads as follows:
“We note several limitations to this study. First, for years with missing smoking prevalence, we interpolated data for 2016 – 2020, and extrapolated data for 2022. For smoking intensity, we interpolated data for 2012 – 2019, and extrapolated data and 2021 and 2022. If our estimates for any of these two variables are higher (lower) than actual numbers, we over-estimate (under-estimate) the quantity of lost revenue. Second, the use of different datasets to estimate smoking behaviour reduces consistency. Datasets from different sources have distinct sampling designs and question wording. Third, self-reported estimates from surveys suffer because of under-reported consumption.6-8 To address under-reporting we scale up consumption by 5% (in the main analysis) and 10% (in the sensitivity analysis). Fourth, the final computation for lost tax revenue is an amalgam of many different estimates and assumptions, each of which is uncertain. To limit this uncertainty, we conducted several sensitivity analyses. Fifth, because we had granular price data for only one year (2017), we used the same adjustment proportion (0.76, using a price elasticity of −0.6) to account for the law of demand in all other years. If the distribution of prices of illicit sales for other years differs from the distribution of prices of illicit sales in 2017, the adjustment proportion would differ from that in 2017. For example, if there is a wide range of prices below the threshold price, the adjustment proportion would be larger than if prices are clustered just below the threshold price. Sixth, National Treasury’s cigarette excise revenue includes revenue from all Southern African Customs Union (SACU) countries (South Africa, Botswana, Lesotho, Eswatini, and Namibia). The number of smokers based on survey data used in our analysis applies only to South Africa. If it were possible to disaggregate

the excise tax revenue data (thereby excluding excise revenue from Botswana, Lesotho, Eswatini, and Namibia), total legal sales in South Africa would be even less than calculated (so illicit trade estimates would be even higher). However, since 94% of smokers in SACU reside in South Africa the impact on the illicit trade is modest. Seventh, it is possible that smokers may have used the money they saved from buying cheaper untaxed cigarettes on other goods that could be subject to excise and VAT (e.g., alcohol). To the extent that this happened, the tax revenue on other products may have increased."

References

1. Vellios N, Ross H, van Walbeek C. The illicit trade of cigarettes in South Africa: 2002−2017. Tobacco Control 2019 doi: 10.1136/tobaccocontrol-2018-054798
2. Filby S, van der Zee K, van Walbeek C. The temporary ban on tobacco sales in South Africa: lessons for endgame strategies. Tobacco Control 2021:tobaccocontrol-2020-056209. doi: 10.1136/tobaccocontrol-2020-056209
3. Crouth G. British American Tobacco fears profits will go up in smoke as illegal trade devours the market. Daily Maverick. https://www.dailymaverick.co.za/article/2023-02-27-british-american-tobacco-fears-profits-will-go-up-in-smoke-as-illegal-trade-devours-the-market/. 27 February 2023
4. Boachie MK, Rossouw L, Ross H. The Economic Cost of Smoking in South Africa, 2016. Nicotine & tobacco research : official journal of the Society for Research on Nicotine and Tobacco 2021;23(2):286-93. doi: 10.1093/ntr/ntaa162 [published Online First: 2020/08/25]
5. World Health Organization. Framework Convention on Tobacco Control. Protocol to Eliminate Illicit Trade in Tobacco Products. http://www.who.int/fctc/protocol/illicit_trade/protocol-publication/en. 2013
6. Roth MA, Aitsi-Selmi A, Wardle H, et al. Under-reporting of tobacco use among Bangladeshi women in England. Journal of Public Health 2009;31(3):326-34. doi: 10.1093/pubmed/fdp060
7. Pérez-Stable EJ, Marín BV, Marín G, et al. Apparent underreporting of cigarette consumption among Mexican American smokers. American journal of public health 1990;80(9):1057-61.
8. Dietz PM, Homa D, England LJ, et al. Estimates of Nondisclosure of Cigarette Smoking Among Pregnant and Nonpregnant Women of Reproductive Age in the United States. American Journal of Epidemiology 2011;173(3):355-59. doi: 10.1093/aje/kwq381
9. U.S. National Cancer Institute and World Health Organization. The Economics of Tobacco and Tobacco Control. National Cancer Institute Tobacco Control Monograph 21. NIH Publication No. 16-CA-8029A. Bethesda, MD: U.S. Department of Health and Human Services, National Institutes of Health, National Cancer Institute; and Geneva, CH: World Health Organization, 2016.
10. International Agency for Research on Cancer. IARC Handbooks of Cancer Prevention, Tobacco Control, Volume 14: Effectiveness of Tobacco Tax and Price Policies for Tobacco Control. Lyon, France, 2011.
11. South African Medical Research Council. Global Adult Tobacco Survey: South Africa, 2021. Factsheet. https://www.health.gov.za/wp-content/uploads/2022/05/Global-Adult-Tobacco-Survey-GATS-SA_FS-Populated__28-April-2022.pdf. 2022
12. International Agency for Research on Cancer. Handbooks of Cancer Prevention, Tobacco Control, Vol. 14: Effectiveness of Tax and Price Policies for Tobacco Control, Lyon, France. 2011
13. Boshoff WH. Cigarette demand in South Africa over 1996-2006: The role of price, income and heaelth awareness. South African Journal of Economics 2008;76(1):118-31. doi: 10.1111/j.1813-6982.2008.00157.x
14. Mukong AK, Tingum EN. The demand for cigarettes: New evidence from South Africa. Development Southern Africa 2019:1-15. doi: 10.1080/0376835x.2019.1640108
15. Van der Zee K, Filby S, Van Walbeek C. When Cigarette Sales Suddenly Become Illegal: Evidence From an Online Survey of South African Smokers During COVID-19 Lockdown. Nicotine & Tobacco Research 2022:ntac067. doi: 10.1093/ntr/ntac067

VERSION 2 – REVIEW

REVIEWER	Ayo-Yusuf, Olalekan<br>Sefako Makgatho Health Sciences University, Dean's office
**REVIEW RETURNED**	03-Dec-2023

**GENERAL COMMENTS**	Major comments:<br>The authors have attempted to respond to some of the reviewer's comments. It was however rather unfortunate that instead of sticking to peer-reviewed published literature, the authors tried to make an assessment and critique of the published literature on RYO. Ironically, the authors chose to rely on 'Personal communication' related to one unpublished survey and several assumptions to justify not changing their estimates of smoking intensity (or running sensitivity) taking account of possible significant RYO among factory-made-cigarette (FMC) smokers.<br>To the extent that the detail of the 2021 survey that the author relied on has not been published and peer-reviewed, there is no evidence that the estimates are any more accurate than those published in previous years. Importantly, it is unlikely that the estimates of RYO cigarette users in 2021 as communicated to authors invalidates estimates published in previous years as obtained from at least 5 waves of SASAS survey in South Africa reported conducted at various times between 2007 and 2018. With the COVID-19 ban, wherein even illicit cigarettes, especially those from non-multinational companies (known to mostly be illicit brands pre-ban) were reported to have been very expensive (see: van der zee et al. https://doi.org/10.1093/ntr/ntac067), I believe RYO might have gained popularity during this period as a price minimization strategy.<br>It is pertinent to note that contrary to the authors' view that questions the accuracy of estimates from the SASAS surveys, a recent published paper adjudged the SASAS surveys that was used to report RYO prevalence over several years as being of low risk of bias while the two surveys the authors relied on for the most of the estimates over the years reported in the current paper, namely AMPS and NIDS, were considered of moderate risk of bias based on an evaluation of 'the wording of the smoking question and the representativeness of the sample' (see attached Table S1 from appendix in: https://www.samedical.org/file/1841).<br><br>In fact, upon closer look at the actual questionnaire used for 2021 GATS in South Africa, it might be a challenge determining prevalence of exclusive current manufactured cigarette smokers using the main question asked.<br>See below the extract question asked to GATS SA survey respondents and the response options below (full questionnaire attached):<br>'I would first like to ask you some questions about smoking tobacco, including manufactured cigarettes, roll your own cigarettes, cigars, cigarillos, pipes, waterpipes (hubbly bubbly) with tobacco. This includes all products where you burn the tobacco as you smoke it. Do you currently smoke tobacco on a daily basis, less than daily, or not at all?<br>DAILY ....<br>LESS THAN DAILY ......<br>NOT AT ALL ....<br>DON'T KNOW...............<br>REFUSED.....................

	Further, the only way to determine smokers of other tobacco products is to isolate the numbers who responded to follow-up questions on number of each of these other products smoked per day. The authors do not seem to have these details too and hence relied on personal communication with the survey's lead researcher for some additional data. This study therefore might be premature and might have not been optimally designed for the questions asked that relies on manufactured cigarette smoking intensity post-2017. It is certainly plausible that some who use a product like RYO and WP (even more common now), but could not estimate their daily use and how long sessions they spent respectively, may skip that question, which will suggest they are not using the product currently (we currently have no information on extent of missing data for the 2021 GATS – estimates on RYO provided to data on unweighted count). Until these estimates from GATS and detail methods are published, it is really not useful to use it as a benchmark to assess accuracy of other published estimates.<br><br>The authors would do well in having an open mind and reading again all the published literature on smoking prevalence in South Africa without bias and, not only read those published by their group. Otherwise, there would be no reason to submit this paper for peer-review if all that would be acceptable are unpublished data (personal communications) and estimates from non-peer reviewed factsheets and own publications of estimates. For example, the authors references one recent published paper (reviewer provided only as example for authors to go search the literature) against the recent unpublished GATS to suggest the past RYO estimates might not be accurate because the estimates from 2018 SASAS might have included small samples. However, the authors ignored another study with much larger pooled samples that have estimated RYO prevalence using pooled 2010 and 2011 surveys (n=6111) compared to the pooled 2017 and 2018 surveys (n=5799) [see: doi: 10.18332/tid/154798], with both samples sizes similar to that of GATS (n~6311). The estimates from the pooled surveys are perhaps more reliable than that from any one survey. It is pertinent to note another paper has also looked at RYO prevalence among smokers as far back as 2007 using similar survey methods making this reliable estimates over long period that the authors study's was also covering.<br><br>The authors also made a comment on a 'strange decrease' in smoking prevalence in 2010 based on the published trend data (Egbe et al. 2023), but then the trend data presented in Table 1 in the paper under review similarly presented a decrease in smoking prevalence from 2007 (25.2%) to 19.8% in 2010 (the lowest in all the years presented). Nonetheless, the authors seem to completely ignore margins of error/levels of uncertainty and take these estimates as absolute/precise numbers.<br><br>In general, the authors seem not to have taken time to read the literature and in particular the paper they have chosen to critique as having 'peculiar' or 'strange' findings based on their own data that have shown illicit trade increase between the same period. As further example, the authors misrepresented findings from the paper by suggesting the paper showed increasing (manufactured) cigarette smoking over this period as a justification for supporting a higher prevalence in 2021. In fact, the trend was that there was no statistically significant increase in manufactured cigarettes smoking even though estimates might have differed between years, it was

	always within the margin of error (i.e. 95% CI). Similarly, if the authors had studied the table they pasted in their response, they would have also observed prevalence RYO use was not significantly different between 2007 and 2017 and the significant difference was between 2017 and 2018. Clearly not only price explains RYO use. For example a landmark court judgement in 2018 decriminalized the private use of cannabis, which is often smoked rolled with tobacco as 'Zol'. So, there might be reasons other than price that may explain the significant increase in RYO in 2018 but that is not the focus here. Importantly, the assumption that illicit cigarette availability has been driving increase in manufactured cigarettes smoking is not supported by data from the study by Egbe et al. (2023) cited by the authors nor supported by authors' own interpolated data (smoking prevalence and smoking intensity seem to have not significantly changed), especially if one considers they did not provide margin of error for their estimates.<br><br>If authors had read literature on smoking prevalence in South Africa, they would also have noted the prevalence of cigarette smoking in 2012 from a large population survey among those ≥18 years was reported at 17.6% (95CI=16.3-18.9)(see Reddy et al. 2015; DOI:10.7196/SAMJnew.7932). The full report (see pg7 in Shisana, et al. 2013; SANHANES-1) also reported the national average smoking intensity as 8.5 CPD, with significantly lower CPD among black Africans (7.6) and residents of urban informal areas (6.7) – both of whom are more likely to use illicit cigarettes (and RYO). The authors' interpolated smoking intensity data for 2012 for 8 million smokers was an average of 9.1 CPD. If authors were to use only just the smoking intensity estimate from SANHANES survey, the illicit prevalence for same number of smokers would be 11.5% (not 14.8%), which is 28.7% lower than their estimate. However, considering the numbers were based of a slightly older age, I expect that the CPD would not be different or in fact lower if it were among those 15-years and older as with the surveys the authors have used. It is important to provide some external validation of interpolation of data and I did not see any.<br><br>Daily use of cigarettes (including RYO and kretek) was reported as 20.5%. However, considering the importance of smoking intensity in the analysis and outcomes, I am still of the view that the authors need to run and present sensitivity analysis not only on the varying demand elasticities, but also based on varying CPD (smoking intensity estimates) considering 20-28% of smokers also smoke RYO (one of the SASAS papers previously demonstrated RYO smokers smoked fewer manufactured CPD ~ 6 CPD). Also consider the fact that only 85-90% of smokers smoke daily (e.g. see: http://dx.doi.org/10.7196/SAMJNEW.7932 and https://www.ajol.info/index.php/safp/article/view/44788/28281 - although dated, but as previously pointed out, the latter reference suggest CPD on days smoked amongst non-daily smokers is about half that of daily smokers ~ 5 vs. 10). In actual fact, I do not think the authors needed to include any analysis based on the prevalence figure of 23.4% over 365 days as not all are daily users of manufactured cigarettes alone. Similarly, I am not convinced about the justification for running sensitivity using an inflation/under reporting figure as high as 10% when the past papers in SA have reported size of illicit consumption based on 5% inflation, just as in table 1 of the current paper.<br><br>Other comments:

	Methods<br>Page 6; lines 37-40: In addressing some of the many assumptions of concern and considering the importance of the size of the base population in estimating illicit cigarette consumption, can the authors clarify to readers what anomalies were found in the UN Population database for just the years 2014-2019 to justify not using it as it used others? Could authors provide estimates using these base population at least as supplement material? The link provided did not quite provide the information.<br>Results<br>Page 8; line 51: it is indicated here that 33.7 billion were self-reported in 2022 but in Table 1, it reflects as 34.7 billion. Which is correct as that influences the estimates (see comment below).<br>Table 1: Last row of last column - the difference 34.7-14.3 = 20.4 NOT 19.4. Therefore, the estimate there would be 58.8% not 57.6% (in any case 19.4/34.7 =55.9% not 57,6%).<br>Table 1: Is there a reason why lower and upper limits are not estimated?<br><br>Discussion<br><br>Page 17; line 29 - the phrase '....legislation WILL be undermined' sounds too authoritative. Yet, other non-price measures have been known to have independent effect on tobacco use, especially in preventing youth from starting. I suggest author consider rather a phrase like '...MAY be undermined'.<br><br>I noted the public health implications have been removed from the conclusion and doesn't appear in the discussion. It is still important to include the public health cost in the paper and perhaps more appropriately in the discussion section.<br><br>Page 17: lines 46-48 - add at the end '...and might be subject to risk for bias'. Then add the reference to the study that has published risk for bias for smoking surveys as a supplement (see: https://www.samedical.org/file/1841).<br><br>It should also be added to the limitations that three out of the five parameters used in determining price threshold of R23 was based on industry own information which might not be reliable given that the industry have been shown to want to overestimate the size of illicit market.

**REVIEWER**	Prieger, James E.<br>Pepperdine University, School of Public Policy
**REVIEW RETURNED**	04-Dec-2023

**GENERAL COMMENTS**	I thank the authors for their responses to all the points raised by the several referees. I especially appreciate the addition of the sensitivity analysis, as I and other commenters suggested.<br><br>Regarding my Comment 3, to which you responded "We are unable to find the first instance where we imply that the ban had no effect on prevalence":<br>On page 3 of your text it reads: "The illicit trade problem was further exacerbated by the five-month Covid-19 related cigarette sales ban in 2020.(6) After the sales ban ended, a substantial proportion of smokers, who had previously smoked multinational brands, stayed with the non-multinational brands. The decrease in

	excise revenue, unaccompanied by a decrease in smoking prevalence, reflects the burgeoning illicit market."<br>I was referring to the claim: "...unaccompanied by a decrease in smoking prevalence..."<br><br>Please address this in the text.

VERSION 2 – AUTHOR RESPONSE

Reviewer: 2

Prof. Olalekan Ayo-Yusuf

Major comments:

Comment:

The authors have attempted to respond to some of the reviewer's comments. It was however rather unfortunate that instead of sticking to peer-reviewed published literature, the authors tried to make an assessment and critique of the published literature on RYO. Ironically, the authors chose to rely on 'Personal communication' related to one unpublished survey and several assumptions to justify not changing their estimates of smoking intensity (or running sensitivity) taking account of possible significant RYO among factory-made-cigarette (FMC) smokers.

To the extent that the detail of the 2021 survey that the author relied on has not been published and peer-reviewed, there is no evidence that the estimates are any more accurate than those published in previous years. Importantly, it is unlikely that the estimates of RYO cigarette users in 2021 as communicated to authors invalidates estimates published in previous years as obtained from at least 5 waves of SASAS survey in South Africa reported conducted at various times between 2007 and 2018.

Response: We apologise for not engaging fully with the RYO literature. We appreciate the time the reviewer took to review the paper a second time. We have taken a deeper look at the four papers suggested by the reviewer. Based on RYO prevalence research, we have changed the sensitivity analysis to account for RYO. In the paragraphs below, we indicate how we have engaged with the literature and how this has affected our results, specifically regarding the size of self-reported manufactured cigarette consumption.

The most helpful RYO paper as it relates to our paper is Prevalence and correlates of roll-your-own cigarette smoking among South African adults during 2010–2011 and 2017–2018

(http://www.tobaccoinduceddiseases.org/Prevalence-and-correlates-of-roll-your-own-cigarette-smoking-namong-South-African,154798,0,2.html)1 as it provides Table 4 (Composition of exclusive RYO cigarette smokers, exclusive manufactured cigarette smokers, and dual smokers of RYO and

manufactured cigarettes, by selected demographic characteristics using pooled data for the four survey cycles combined (2010, 2011, 2017, and 2018)). Table 4 categorises smokers into three mutually exclusive groups: exclusive RYO cigarette smokers, exclusive manufactured cigarette smokers, and dual smokers (RYO and manufactured cigarette smokers). We use this information to downscale our estimates of self-reported manufactured cigarette consumption in the sensitivity analysis. The following table provides a summary of how we got to □10%, using the example of 100 smokers:

Number of smokers from combined datasets (SASAS 2010, 2011, 2017, and 2018)1

% of total smokers Manufactured cigarettes per day per smoker Sticks per day for 100 people (smokers*manufactured cigarettes per day per smoker)

Exclusive RYO smokers 128 5.2 0 0

Dual smokers 515 21.1 6 127

Exclusive manufactured cig smokers 1803 73.7 9 663

Total 2446 100.0 15 790

The number of 9 cigarettes per day for exclusive manufactured cigarette smokers is the average number of cigarettes for the period 2010 to 2018 (from our time series data). We assume that dual smokers smoke 6 manufactured cigarettes per day and 3 RYO cigarettes a day. The total number of manufactured cigarettes smoked by these 100 people is 790 cigarettes.

Now we compare 790 cigarettes to the number we used before we considered RYO use. Previously, we assumed that all 100 smokers smoked 9 manufactured cigarettes per day. This gives a total of 900 cigarettes.

The percentage difference between the old (not accounting for RYO smokers) and the new method (accounting for RYO smokers) is □12.2% ((790□900/900)*100).

We repeat the same exercise using the GATS 2021 data:

Ignoring kretek:

Exclusive RYO smokers 0.5% This is 23.9% less 23.4%

Dual smokers 2.3% This is 23.9% less 0.5% (exclusive RYO smokers) less 21.1% (exclusive manufactured cigarette smokers)

Exclusive manufactured cig smokers 21.1% This is 23.9% less 2.8%

Any cigarettes 23.9%

Prevalence % of total smokers Manufactured cigarettes per day per smoker Sticks per day for 100 people (smokers*manufactured cigarettes per day per smoker)

Exclusive RYO 0.5% 2.1 0 0

Dual users 2.3% 9.6 6 58

Exclusive manufactured cigs 21.1% 88.3 9 795

Total 23.9% 100.0 15 852

The percentage difference between the old and the new method is □5.3% ((852□900/900)*100).

Taking the average of the two percentage differences (□12.2 and □5.3) is □8.8%. We round to □10%.

We use the +5% under-reporting as the base. +5% plus □10% is □5%. In our initial submission, we adjusted the self-reported number of cigarettes by +5% (baseline) and +10% (for the sensitivity analysis). We have now adjusted the self-reported number of cigarettes by +5% (baseline) and □5% (sensitivity analysis; lower bound; assuming that RYO cigarettes comprise 10% of the total number of cigarettes consumed).

The text now reads as follows:

“To account for the uncertainty around the various assumptions, we conducted several sensitivity analyses. We varied the following parameters: (1) price elasticity (□□□□, □□□6, and □□□8), (2) smoking prevalence for 2021 (20.0% and 23.4%), and the interpolated values for the period 2015 to 2020, and (3) under- and over-reporting (5% □5%), which results in 12 combinations. Over-reporting of □5% (the negative sign in effect means that we assume that cigarette consumption was over-reported) was chosen to account for the presence roll-your-own (RYO) cigarettes. RYO cigarette prevalence has been increasing in South Africa,2 and it is possible that respondents included the use of RYO when they were asked about smoking. Combined datasets from 2010 to 2018, indicates that the percentage of smokers who were exclusive RYO smokers was 5.2%, dual smokers (manufactured cigarettes and RYO) was 21.1%, and exclusive manufactured cigarettes smokers was 73.7%.1 We consider 5% under-reporting (upper bound) as the baseline. Assuming that RYO cigarettes comprise 10% of the total number of cigarettes consumed, we apply an over-reporting

adjustment of □5% (sensitivity analysis; lower bound) from 2010 to 2022 (a decrease of □5% before 2010 results in nonsensical negative illicit trade estimates).”

As a result of this adjustment, the sensitivity analysis changes substantially, resulting in reduced estimates of lost revenue for the various scenarios.

Table 2: Sensitivity analysis (Before)

Table 2: Sensitivity analysis (After)

We have changed the main findings:

Before:

The lowest lost revenue estimate for the period 2002 to 2022 is R91.7 billion (in 2022 prices) (elasticity:□□□8, smoking prevalence in 2021: 20%, under-reporting: 5%), while the highest is R157.0 billion (2022 prices) (elasticity:□□□4, smoking prevalence in 2021: 23.4%, under-reporting: 10%) (Table 2).

After:

The lowest lost revenue estimate for the period 2002 to 2022 is R65.3 billion (in 2022 prices) (elasticity:□□□8, smoking prevalence in 2021: 20%, over-reporting: □5%), while the highest is R129.7.0 billion (2022 prices) (elasticity:□□□4, smoking prevalence in 2021: 23.4%, under-reporting: 5%) (Table 2).

Comment:

With the COVID-19 ban, wherein even illicit cigarettes, especially those from non-multinational companies (known to mostly be illicit brands pre-ban) were reported to have been very expensive (see: van der zee et al. https://doi.org/10.1093/ntr/ntac067), I believe RYO might have gained popularity during this period as a price minimization strategy.

Response:

In the first online study our research group did on the sales ban smoking behaviour, we asked respondents to provide us with “any further comments” about the sales ban. Of the 12 204 respondents, 5 831 provided additional comments. These comments were analysed and categorised. The comments covered a lot of issues, but we did not pick up that respondents were switching to RYO tobacco. Despite not finding evidence, and in light of the sharp increases in cigarette prices during the sales ban, it seems intuitive that there would be an increase in RYO tobacco. We hope that the new sensitivity analysis goes some way in including RYO.

Comment:

It is pertinent to note that contrary to the authors’ view that questions the accuracy of estimates from the SASAS surveys, a recent published paper adjudged the SASAS surveys that was used to report RYO prevalence over several years as being of low risk of bias while the two surveys the authors relied on for the most of the estimates over the years reported in the current paper, namely AMPS and NIDS, were considered of moderate risk of bias based on an evaluation of 'the wording of the smoking question and the representativeness of the sample' (see attached Table S1 from appendix in: https://www.samed]ical.org/file/1841).

Response:

This is a good point and not one we had previously considered. One of the reasons why NIDS scored low on the risk of bias assessment was that NIDS was designed as a panel survey, so the assessors weren’t sure if the waves subsequent to wave 1 were nationally representative. NIDS econometricians have ensured that using the NIDS waves as cross-sections is valid. We are fortunate to be in the same building as the NIDS team so over the years they have been instrumental in helping us with the data. The AMPS datasets scored low because the information is not readily available from the south African Audience Research Foundation (SAARF) (private company), unlike data collected by academic institutions.

Comment:

In fact, upon closer look at the actual questionnaire used for 2021 GATS in South Africa, it might be a challenge determining prevalence of exclusive current manufactured cigarette smokers using the main question asked.

See below the extract question asked to GATS SA survey respondents and the response options below:

'I would first like to ask you some questions about smoking tobacco, including manufactured cigarettes, roll your own cigarettes, cigars, cigarillos, pipes, waterpipes (hubbly bubbly) with tobacco. This includes all products where you burn the tobacco as you smoke it.

Do you currently smoke tobacco on a daily basis, less than daily, or not at all?

DAILY ....

LESS THAN DAILY ......

NOT AT ALL ....

DON'T KNOW..............

REFUSED....................

Further, the only way to determine smokers of other tobacco products is to isolate the numbers who responded to follow-up questions on number of each of these other products smoked per day. The authors do not seem to have these details too and hence relied on personal communication with the survey's lead researcher for some additional data. This study therefore might be premature and might have not been optimally designed for the questions asked that relies on manufactured cigarette smoking intensity post-2017. It is certainly plausible that some who use a product like RYO and WP (even more common now), but could not estimate their daily use and how long sessions they spent respectively, may skip that question, which will suggest they are not using the product currently (we currently have no information on extent of missing data for the 2021 GATS – estimates on RYO provided to data on unweighted count). Until these estimates from GATS and detail methods are published, it is really not useful to use it as a benchmark to assess accuracy of other published estimates.

Response:

The GATS surveys follow a framework and methods used in more than 30 low- and middle-income countries. For GATS SA 2021, a total of 7245 households were sampled and one individual was randomly selected from each participating household to complete the survey (n=6,311 completed individual interviews; overall response rate of 91.5%). We have no reason to believe that the GATS 2021 prevalence estimates, both the online factsheet and the RYO prevalence we received from a Specialist Scientist at the SA Medical Research Council, is unreliable.

Regarding determining prevalence of exclusive current manufactured cigarette smokers, other questions in the GATS survey include:

B06. On average, how many of the following products do you currently smoke each day? Also, let me know if you smoke the product, but not every day.

B08. How many of the following do you currently smoke during a usual week?

We agree that using unweighted data would be problematic. However, the data we use is indeed weighted. The first snippet below is from the GATS factsheet, showing that the prevalence of current cigarette smokers is 23.9%. The footnotes on the factsheet state that "Data have been weighted to be nationally representative of all non-institutionalized men and women aged 15 years and older. Percentages reflect the prevalence of each indicator in each group, not the distribution across groups". The first column indicates the overall prevalence (i.e. men and women combined), the second column shows the prevalence among men and the third column the prevalence among women. The second snippet is the data we received from the MRC. The prevalence of any cigarette smoking is 23.9% (for males and females), corresponding with the weighted prevalence percentage from the factsheet.

Comment:

The authors would do well in having an open mind and reading again all the published literature on smoking prevalence in South Africa without bias and, not only read those published by their group. Otherwise, there would be no reason to submit this paper for peer-review if all that would be acceptable are unpublished data (personal communications) and estimates from non-peer reviewed factsheets and own publications of estimates. For example, the authors references one recent published paper (reviewer provided only as example for authors to go search the literature) against the recent unpublished GATS to suggest the past RYO estimates might not be accurate because the estimates from 2018 SASAS might have included small samples. However, the authors ignored another study with much larger pooled samples that have estimated RYO prevalence using pooled 2010 and 2011 surveys (n=6111) compared to the pooled 2017 and 2018 surveys (n=5799) [see: doi: 10.18332/tid/154798], with both samples sizes similar to that of GATS (n~6311). The estimates from the pooled surveys are perhaps more reliable than that from any one survey. It is pertinent to note another paper has also looked at RYO prevalence among smokers as far back as 2007 using similar survey methods making this reliable estimates over long period that the authors study's was also covering.

Response:

We have adjusted our estimates downwards to account for RYO users (see the discussion on the first substantial point above).

Comment:

The authors also made a comment on a ‘strange decrease’ in smoking prevalence in 2010 based on the published trend data (Egbe et al. 2023), but then the trend data presented in Table 1 in the paper under review similarly presented a decrease in smoking prevalence from 2007 (25.2%) to 19.8% in 2010 (the lowest in all the years presented). Nonetheless, the authors seem to completely ignore margins of error/levels of uncertainty and take these estimates as absolute/precise numbers.

Response:

We agree that the same decrease in 2010 is evident in AMPS. We apologise for making this error.

Comment:

In general, the authors seem not to have taken time to read the literature and in particular the paper they have chosen to critique as having ‘peculiar’ or ‘strange’ findings based on their own data that have shown illicit trade increase between the same period. As further example, the authors misrepresented findings from the paper by suggesting the paper showed increasing (manufactured) cigarette smoking over this period as a justification for supporting a higher prevalence in 2021. In fact, the trend was that there was no statistically significant increase in manufactured cigarettes smoking even though estimates might have differed between years, it was always within the margin of error (i.e. 95% CI). Similarly, if the authors had studied the table they pasted in their response, they would have also observed prevalence RYO use was not significantly different between 2007 and 2017 and the significant difference was between 2017 and 2018. Clearly not only price explains RYO use. For example a landmark court judgement in 2018 decriminalized the private use of cannabis, which is often smoked rolled with tobacco as ‘Zol’. So, there might be reasons other than price that may explain the significant increase in RYO in 2018 but that is not the focus here. Importantly, the assumption that illicit cigarette availability has been driving increase in manufactured cigarettes

smoking is not supported by data from the study by Egbe et al. (2023) cited by the authors nor supported by authors' own interpolated data (smoking prevalence and smoking intensity seem to have not significantly changed), especially if one considers they did not provide margin of error for their estimates.

Response:

We have studied this comment carefully, and the published papers on the prevalence of RYO and manufactured cigarettes. Our understanding is that the reviewer's main point is that the prevalence of manufactured cigarettes has not increased substantially, while the prevalence of RYO has increased over the past number of years.

We have been transparent in explaining that we have had to interpolate a number of variables, because the raw data were not available. In order to make this more explicit, we have added 95% confidence for the relevant years in Table 1. Where we interpolated numbers, we are unable to show the confidence intervals.

Although the percentage increase in smoking prevalence is small, it translates to a substantial increase in the absolute number of smokers. In 2018, we estimated that there were 9.2 million smokers, increasing to 10.0 million smokers in 2022.

Comment:

If authors had read literature on smoking prevalence in South Africa, they would also have noted the prevalence of cigarette smoking in 2012 from a large population survey among those ≥18 years was reported at 17.6% (95CI=16.3-18.9)(see Reddy et al. 2015; DOI:10.7196/SAMJnew.7932). The full report (see pg7 in Shisana, et al. 2013; SANHANES-1) also reported the national average smoking intensity as 8.5 CPD, with significantly lower CPD among black Africans (7.6) and residents of urban informal areas (6.7) – both of whom are more likely to use illicit cigarettes (and RYO). The authors' interpolated smoking intensity data for 2012 for 8 million smokers was an average of 9.1 CPD. If authors were to use only just the smoking intensity estimate from SANHANES survey, the illicit prevalence for same number of smokers would be 11.5% (not 14.8%), which is 28.7% lower than their estimate. However, considering the numbers were based of a slightly older age, I expect that the CPD would not be different or in fact lower if it were among those 15-years and older as with the surveys the authors have used. It is important to provide some external validation of interpolation of data and I did not see any.

Response:

Given that the present study is a time series study, we aimed, wherever possible, to use data that were collected in multiple rounds/waves, using a consistent methodology. From our understanding, SANHANES was a once-off survey. The fact that SANHANES yielded smoking prevalence estimates that were substantially different from other sources, puzzles us.

Regarding doing a sensitivity analysis using the SANHANES 2012 figure of 8.5 cigarettes per day (instead of 9.1 as we use), the new sensitivity analysis (-5% versus +5%) account for lower self-reported consumption.

Comment:

Daily use of cigarettes (including RYO and kretek) was reported as 20.5%. However, considering the importance of smoking intensity in the analysis and outcomes, I am still of the view that the authors need to run and present sensitivity analysis not only on the varying demand elasticities, but also based on varying CPD (smoking intensity estimates) considering 20-28% of smokers also smoke RYO (one of the SASAS papers previously demonstrated RYO smokers smoked fewer manufactured CPD ~ 6 CPD). Also consider the fact that only 85-90% of smokers smoke daily (e.g. see: http://dx.doi.org/10.7196/SAMJNEW.7932 and https://www.ajol.info/index.php/safp/article/view/44788/28281 - although dated, but as previously pointed out, the latter reference suggest CPD on days smoked amongst non-daily smokers is about half that of daily smokers ~ 5 vs. 10). In actual fact, I do not think the authors needed to include any analysis based on the prevalence figure of 23.4% over 365 days as not all are daily users of manufactured cigarettes alone.

Response:

See above regarding updated numbers that downscale self-reported consumption.

We agree that non-daily smokers have a smaller CPD. Overall CPD is an average figure which includes the total number of cigarettes smoked by daily and non-daily smokers. If the average CPD across all smokers is 9 CPD, and 15% of smokers are non-daily smokers, and the average CPD among non-daily smokers is 6 CPD, then the average CPD of the daily smokers would be [(9 x 100) – (6 x 15)]/85 = 9.53.

We have graphed prevalence data from several surveys. The trends of SASAS, NIDS, and AMPS suggest that cigarette smoking prevalence is increasing (although not significant). Based on the trend analysis, we prefer to keep 23.4% in the main analysis as the prevalence number for 2021. In the sensitivity analysis, we reran the analysis using a smoking prevalence of 20% in 2021.

Comment:

Similarly, I am not convinced about the justification for running sensitivity using an inflation/under reporting figure as high as 10% when the past papers in SA have reported size of illicit consumption based on 5% inflation, just as in table 1 of the current paper.

Response:

We have deleted 10% under-reporting and replaced it with □5%, i.e. over-reporting of 5%. Please see table 2 above.

Other comments:

Methods

Comment:

Page 6; lines 37-40: In addressing some of the many assumptions of concern and considering the importance of the size of the base population in estimating illicit cigarette consumption, can the

authors clarify to readers what anomalies were found in the UN Population database for just the years 2014-2019 to justify not using it as it used others? Could authors provide estimates using these base population at least as supplement material? The link provided did not quite provide the information.

Response:

The below table and figure below shows the changes we made. The numbers in bold are the numbers that are implausible and that we subsequently smoothed.

Year Original data Growth rate (%) (Original data) Smoothed data Growth rate (%) (Smoothed data) Difference (absolute numbers) Difference between original & smoothed data (%) (absolute numbers)

2002 31 822 721 31 822 721 0 0,00

2003 32 576 493 2,4 32 576 493 2,4 0 0,00

2004 33 293 877 2,2 33 293 877 2,2 0 0,00

2005 33 955 649 2,0 33 955 649 2,0 0 0,00

2006 34 573 105 1,8 34 573 105 1,8 0 0,00

2007 35 169 345 1,7 35 169 345 1,7 0 0,00

2008 35 748 666 1,6 35 748 666 1,6 0 0,00

2009 36 321 566 1,6 36 321 566 1,6 0 0,00

2010 36 904 086 1,6 36 904 086 1,6 0 0,00

2011 37 485 850 1,6 37 485 850 1,6 0 0,00

2012 38 036 829 1,5 38 036 829 1,5 0 0,00

2013 38 560 714 1,4 38 560 714 1,4 0 0,00

2014 39 189 156 1,6 39 038 104 1,2 -151 052 0,39

2015 40 077 226 2,3 39 515 494 1,2 -561 732 1,42

2016 40 417 614 0,8 39 992 885 1,2 -424 729 1,06

2017 40 449 699 0,1 40 470 275 1,2 20 576 -0,05

2018 40 893 751 1,1 40 947 665 1,2 53 915 -0,13

2019 41 389 775 1,2 41 425 055 1,2 35 281 -0,09

2020 41 902 446 1,2 41 902 446 1,2 0 0,00

2021 42 362 736 1,1 42 362 736 1,1 0 0,00

2022 42 883 798 1,2

We believe that the smoothed data is a better fit of population changes. There is no reason to believe that the population growth rate spiked in 2014, 2015, and 2016, and then dropped dramatically in 2017.

Given the above, we do not think it would add value to provide additional estimates using the unsmoothed numbers.

Results

Comment:

Page 8; line 51: it is indicated here that 33.7 billion were self-reported in 2022 but in Table 1, it reflects as 34.7 billion. Which is correct as that influences the estimates (see comment below).

Response:

Thank you for picking up this typo. The typo was in the table (we have changed from 34.7 to 33.7). We have checked that there aren't any other errors in the table.

Comment:

Table 1: Last row of last column - the difference 34.7-14.3 = 20.4 NOT 19.4. Therefore, the estimate there would be 58.8% not 57.6% (in any case 19.4/34.7 =55.9% not 57,6%).

Response:

Resolved by previous comment.

Comment:

Table 1: Is there a reason why lower and upper limits are not estimated?

Response:

Table 1 now includes 95% CIs. We have added this text to the paper:

"To create confidence intervals around annual self-reported cigarette consumption, Stata version 16.0 was used to bootstrap the point estimate of the product of individual smoking status (which is used to determine smoking prevalence in the population) and smoking intensity. Bootstrapping obtains a valid standard error by computing the estimate from different random samples drawn from the original data.3 We used the same bootstrap method as Paraje 4 to estimate the confidence intervals.

Smoking status is a discrete variable (1: smoker, 0: non-smoker), while cigarette consumption is a continuous variable. Respondents who reported that they smoked, but did not report the number of cigarettes they smoked per day, were assigned the mean value of smoking intensity in that year.

Where we did not have smoking intensity data (AMPS 2012 − 2015), we only bootstrapped the number of smokers, assuming cigarette smoking intensity estimates as presented in Table 1.

Since the bootstrap command does not allow weights to be accounted for using the svy command, we multiplied smoking*intensity by the weight variable before bootstrapping. All missing values were dropped before running the bootstrap command (failing to do so resulted in error messages). We specified 1000 repetitions in the bootstrap command.

Once the point estimate was obtained, it was multiplied by 365, and by the total number of respondents in each respective survey. For example, in AMPS 2002, annual self-reported consumption was 26.5 billion cigarettes (2466 cigarettes per day (bootstrap point estimate) * 365 (days per year) * 29 458 (number of respondents)). To obtain the 95% confidence intervals for annual self-reporting cigarette consumption, the same formula was applied, using the lower and upper bound estimates."

Discussion

Comment:

Page 17; line 29 - the phrase '....legislation WILL be undermined' sounds too authoritative. Yet, other non-price measures have been known to have independent effect on tobacco use, especially in preventing youth from starting. I suggest author consider rather a phrase like '...MAY be undermined'.

Response:

Thanks for the suggestion. We have made the change.

Comment:

I noted the public health implications have been removed from the conclusion and doesn't appear in the discussion. It is still important to include the public health cost in the paper and perhaps more appropriately in the discussion section.

Response:

Thank you for this suggestion. We have included the following paragraph in the discussion:

'A 2021 paper 5 found that the economic cost of smoking in South Africa in 2016 was R42 billion (US$2.88 billion), of which R14.48 billion was for healthcare costs (hospitalization and outpatient department visits).'

Comment:

Page 17: lines 46-48 - add at the end '...and might be subject to bias'. Then add the reference to the study that has published risk for bias for smoking surveys as a supplement (see: https://www.samedical.org/file/1841).

Response:

Done.

Comment:

It should also be added to the limitations that three out of the five parameters used in determining price threshold of R23 was based on industry own information which might not be reliable given that the industry have been shown to want to overestimate the size of illicit market.

Response:

We have added this sentence: “Eighth, three out of the five parameters used to determine the price threshold of R23 were based on information from the tobacco industry, which may be unreliable. However, these three parameters comprise about 30% of the threshold price, so even if there are, they should have only a modest impact on the magnitude of the threshold price.”

Overall response:

We thank the reviewer for helping us improve the paper substantially.

Reviewer: 3

Dr. James E. Prieger, Pepperdine University

Comment:

I thank the authors for their responses to all the points raised by the several referees. I especially appreciate the addition of the sensitivity analysis, as I and other commenters suggested.

Response:

Thank you.

Comment:

Regarding my Comment 3, to which you responded "We are unable to find the first instance where we imply that the ban had no effect on prevalence":

On page 3 of your text it reads: "The illicit trade problem was further exacerbated by the five-month Covid-19 related cigarette sales ban in 2020.(6) After the sales ban ended, a substantial proportion of smokers, who had previously smoked multinational brands, stayed with the non-multinational brands. The decrease in excise revenue, unaccompanied by a decrease in smoking prevalence, reflects the burgeoning illicit market."

I was referring to the claim: "...unaccompanied by a decrease in smoking prevalence..."

Response:

Thanks you for the clarification. We have changed the sentence to: “The decrease in excise revenue, if unaccompanied by a decrease in smoking prevalence, indicates a growing illicit market.”

References

1. Nkosi L, Agaku IT, Ayo-Yusuf O. Prevalence and correlates of roll-your-own cigarette smoking among South African adults during 2010–2011 and 2017–2018. Tob Induc Dis 2022;20(November):1-12. doi: 10.18332/tid/154798

2. Egbe CO, Gwambe S, Londani M, et al. Trends in the use and dual use of factory-made combustible cigarettes, other tobacco products and electronic cigarettes: Results from South African Social Attitudes Surveys during 2007 to 2018. Tob Induc Dis 2023;21(July):1-13. doi: 10.18332/tid/168121

3. Wooldridge JM. Introductory Econometrics: A Modern Approach. 5th edition ed. USA: South-Western, Cengage Learning 2013.

4. Paraje G. Illicit Cigarette Trade in Five South American Countries: A Gap Analysis for Argentina, Brazil, Chile, Colombia and Peru. Nicotine & Tobacco Research 2018 doi: 10.1093/ntr/nty098

5. Boachie MK, Rossouw L, Ross H. The Economic Cost of Smoking in South Africa, 2016. Nicotine & tobacco research : official journal of the Society for Research on Nicotine and Tobacco 2021;23(2):286-93. doi: 10.1093/ntr/ntaa162 [published Online First: 2020/08/25]

VERSION 3 – REVIEW

**REVIEWER**	Ayo-Yusuf, Olalekan<br>Sefako Makgatho Health Sciences University, Dean's office
**REVIEW RETURNED**	19-Feb-2024

**GENERAL COMMENTS**	I commend the authors for significant improvement in the paper and in responding to the reviewers' comments. I however still have few but important comments for consideration of the authors.<br><br>Abstract

	Lines 55-57<br>I strongly recommend that the abstract reflects somewhere the fact that sensitivity analysis as requested by more than one reviewer was done and the results should be indicated as a range in addition to main results estimate. For example, authors may consider something along the lines of .... 'Based on sensitivity analysis, which took into account varying assumptions made in estimating lost revenue, it was estimated that from 2002 to 2022, government lost R119bilion (Range R65 - R129 billion) in excise and VAT revenues.'<br><br>Same could be reflected in the main text results and in the Discussion. Otherwise, the sensitivity analysis has not meaning as it currently stands as it is not reflected anywhere else except in the table.<br><br>Page 14; Table 1:<br>line 41; last column: The number '6.2' might have been erroneously entered with the other numbers. The authors should recheck and make corrections accordingly.<br><br>Table 1; Line 50; last column: Similar to others with similar parameters available up to 2015, the authors should also report the 95% CI for illicit trade estimates for 2021, at the very least.

VERSION 3 – AUTHOR RESPONSE

Reviewer: 2
Prof. Olalekan Ayo-Yusuf, Sefako Makgatho Health Sciences University, Harvard School of Public Health

Comments to the Author: I commend the authors for significant improvement in the paper and in responding to the reviewers' comments. I however still have few but important comments for consideration of the authors.

Response: Thank you.

Comment 1: Abstract. Lines 55-57. I strongly recommend that the abstract reflects somewhere the fact that sensitivity analysis as requested by more than one reviewer was done and the results should be indicated as a range in addition to main results estimate. For example, authors may consider something along the lines of .... 'Based on sensitivity analysis, which took into account varying assumptions made in estimating lost revenue, it was estimated that from 2002 to 2022, government lost R119bilion (Range R65 - R129 billion) in excise and VAT revenues.'

Same could be reflected in the main text results and in the Discussion. Otherwise, the sensitivity analysis has not meaning as it currently stands as it is not reflected anywhere else except in the table.

Response: We have added this sentence in the results of the abstract, and to the discussion: "A comprehensive sensitivity analysis indicates that the estimated lost revenue of R119 billion from 2002 to 2022 falls within the range of R65 billion to R130 billion (all 2022 prices)."

Comment: Page 14; Table 1: line 41; last column: The number '6.2' might have been erroneously entered with the other numbers. The authors should recheck and make corrections accordingly.

Response: Done

Comment: Table 1; Line 50; last column: Similar to others with similar parameters available up to 2015, the authors should also report the 95% CI for illicit trade estimates for 2021, at the very least.

Response: Unfortunately we are not able to add in 95% CIs to the illicit trade estimates as we do not have 95% CIs around smoking prevalence and smoking intensity.

Reviewer: 2
Competing interests of Reviewer: I have no competing interest to declare
